# Molecular cytogenetics and development of St-chromosome-specific molecular markers of novel stripe rust resistant wheat–*Thinopyrum intermedium* and wheat–*Thinopyrum ponticum* substitution lines

**DOI:** 10.1186/s12870-022-03496-x

**Published:** 2022-03-12

**Authors:** Siwen Wang, Changyou Wang, Xianbo Feng, Jixin Zhao, Pingchuan Deng, Yajuan Wang, Hong Zhang, Xinlun Liu, Tingdong Li, Chunhuan Chen, Baotong Wang, Wanquan Ji

**Affiliations:** 1 College of Agronomy, Northwest A&F University, Yangling, 712100 Shaanxi China; 2 Shaanxi Research Station of Crop Gene Resources and Germplasm Enhancement, Ministry of Agriculture, Yangling, 712100 Shaanxi China; 3 State Key Laboratory of Crop Stress Biology for Arid Areas, Yangling, 712100 Shaanxi China; 4 College of Plant Protection, Northwest A&F University, Yangling, 712100 Shaanxi China

**Keywords:** *Thinopyrum intermedium*, *Thinopyrum ponticum*, Substitution lines, Stripe rust resistance, St-chromosome-specific molecular markers

## Abstract

**Background:**

Owing to their excellent resistance to abiotic and biotic stress, *Thinopyrum intermedium* (2*n* = 6*x* = 42, JJJ^s^J^s^StSt) and *Th. ponticum* (2*n* = 10*x* = 70) are both widely utilized in wheat germplasm innovation programs. Disomic substitution lines (DSLs) carrying one pair of alien chromosomes are valuable bridge materials for transmission of novel genes, fluorescence in situ hybridization (FISH) karyotype construction and specific molecular marker development.

**Results:**

Six wheat–*Thinopyrum* DSLs derived from crosses between Abbondanza nullisomic lines (2*n* = 40) and two octoploid *Trititrigia* lines (2*n* = 8*x* = 56), were characterized by sequential FISH–genome in situ hybridization (GISH), multicolor GISH (mc-GISH), and an analysis of the wheat 15 K SNP array combined with molecular marker selection. ES-9 (DS2St (2A)) and ES-10 (DS3St (3D)) are wheat–*Th. ponticum* DSLs, while ES-23 (DS2St (2A)), ES-24 (DS3St (3D)), ES-25(DS2St (2B)), and ES-26 (DS2St (2D)) are wheat–*Th. intermedium* DSLs. ES-9, ES-23, ES-25 and ES-26 conferred high thousand-kernel weight and stripe rust resistance at adult stages, while ES-10 and ES-24 were highly resistant to stripe rust at all stages. Furthermore, cytological analysis showed that the alien chromosomes belonging to the same homoeologous group (2 or 3) derived from different donors carried the same FISH karyotype and could form a bivalent. Based on specific-locus amplified fragment sequencing (SLAF-seq), two 2St-chromosome-specific markers (PTH-005 and PTH-013) and two 3St-chromosome-specific markers (PTH-113 and PTH-135) were developed.

**Conclusions:**

The six wheat–*Thinopyrum* DSLs conferring stripe rust resistance can be used as bridging parents for transmission of valuable resistance genes. The utility of PTH-113 and PTH-135 in a BC1F2 population showed that the newly developed markers could be useful tools for efficient identification of St chromosomes in a common wheat background.

**Supplementary Information:**

The online version contains supplementary material available at 10.1186/s12870-022-03496-x.

## Background

Intermediate wheatgrass (*Thinopyrum intermedium* Barkworth & D.R. Dewey, JJJ^s^J^s^StSt, 2*n* = 6*x* = 42) and tall wheatgrass (*Th. ponticum* (Podp.) Barkworth & D. R. Dewey, 2*n* = 10*x* = 70) are important allopolyploids of *Thinopyrum* species. Because of their desirable tolerance to biotic and abiotic stresses, both have been widely used in wheat chromosome engineering for decades [1, 2]. The chromosomal composition of *Th. intermedium* and *Th. ponticum* have not been fully characterized. For *Th. intermedium*, the chromosomal composition is generally regarded as JJJ^s^J^s^StSt [3] or J^r^J^r^J^vs^J^vs^StSt [4]. The subgenome J or J^r^ is highly homologous with genome J (*Th. bessarabicum*, J^b^, E^b^)/E (*Th. elongatum*, J^e^, E^e^) [5], and the main controversy has been whether genome V originating from *Dasypyrum villosum* (2*n* = 2*x* = 14, VV) was involved in the recombinant subgenome J^s^ [6, 7]. Additionally, it was determined that *Th. intermedium* contained a set of St chromosomes probably derived from diploid *Pseudoroegneria spicata* or *P. strigosa* (2*n* = 2*x* = 14, StSt) [8–10]. However, it is still unclear whether *Th. ponticum* contains St chromosomes and if the St genome or the J/E genome were affected by recombination during the allopolyploidization process [11, 12].

Although aspects of the *Th. intermedium* and *Th. ponticum* genomes remain undiscovered, numerous partial amphiploid lines have been successfully developed [13–18]. Octoploid *Trititrigia* with advantageous traits is a significant cytogenetic resource for developing alien introgression lines that can be further applied to wheat breeding programs [19–23]. Generally, octoploid *Trititrigia* lines carry a synthetic genome inherited from *Th. intermedium* or *Th. ponticum*. Therefore, the chromosomal compositions of *Thinopyrum* allopolyploids can be understood according to the defined genome compositions of partial amphiploids based on molecular cytogenetic methods. TAF46 is an important wheat–*Th. intermedium* partial amphiploid with a common wheat Vilmorin 27 background, and six disomic addition lines (DALs), L1, L2, L3, L4, L5 and L7, were developed from TAF46 [24]. Subsequently, molecular cytogenetic identification of TAF46 as well as the six derived DALs revealed that the genome composition of TAF46 was 14A + 14B + 14D + 2(1 J) + 2(2St) + 2(3 J) + 2(4St) + 2(5 J) + 2(6St) + 2(7 J) [25–27], which suggested that chromosomes of the St genome contained in *Th. intermedium* could be stably inherited, and that it is feasible to introduce the St chromosomes into a common wheat background for wheat genetic improvement.

Stripe rust (*Puccinia striiformis* f.sp*. tritici*, *Pst*) is a recurrent disease that causes serious annual decreases in wheat yields [28, 29]. Development and transfer of novel resistance genes contained in related wild wheat species is one of the most efficient and environmentally-friendly approaches to fighting stripe rust. According to previous studies, St chromosomes originating from *Th. intermedium* carry several new stripe rust resistance genes, which are potentially optimal genetic resources for wheat breeding. In addition to the named wheat–*Th. intermedium* DALs, L4 (DA4St) and L7 (DA6St), a DS1St (1D) with stripe rust resistance was produced [30]. Moreover, a DA3St [31] and a DA7St [32] were characterized, both carrying stripe rust resistance gene(s). In our previous study, ES-12 (DS3St (3D)) containing chromosome 3St derived from *Th. ponticum* also conferred stripe rust resistance [33]. However, at present no wheat–*Thinopyrum* 2St disomic substitution lines (DSLs) with stripe rust resistance have been reported.

Xiaoyan784 and Zhong4 are both significant octoploid *Trititrigia* lines conferring stripe rust resistance. Xiaoyan784 (2*n* = 8*x* = 56) was produced from distant hybridization between common wheat and *Th. ponticum* [34], while Zhong4 (2*n* = 8*x* = 56) was developed from distant hybridization between common wheat and *Th. intermedium* by Sun in 1965 [35]. Abbondanza nullisomic lines (2*n* = 40) were developed by Xue in 1991, and have been used as valuable plant materials to efficiently create substitution alien lines for several decades [36]. In the present study, four wheat–*Th. intermedium* DSLs, ES-22, ES-23, ES-25, and ES-26, were generated from crosses between Abbondanza nullisomic lines and Zhong4 with consecutive self-crosses for several years. Two wheat–*Th. ponticum* DSLs, ES-9 and ES-10, were derived from Xiaoyan784.Molecular cytogenetic analysis was used to determine and compare the genome compositions of the six alien lines. In addition, stripe rust resistance and potential value of the morphological characteristics for wheat breeding were evaluated. Finally, St-chromosome-specific markers were developed by specific-locus amplified fragment sequencing (SLAF-seq) and validated. These markers could be useful tools for efficient identification of St chromosomes in a common wheat background.

## Results

### In situ hybridization of the six substitution lines

**Fig. 1:**
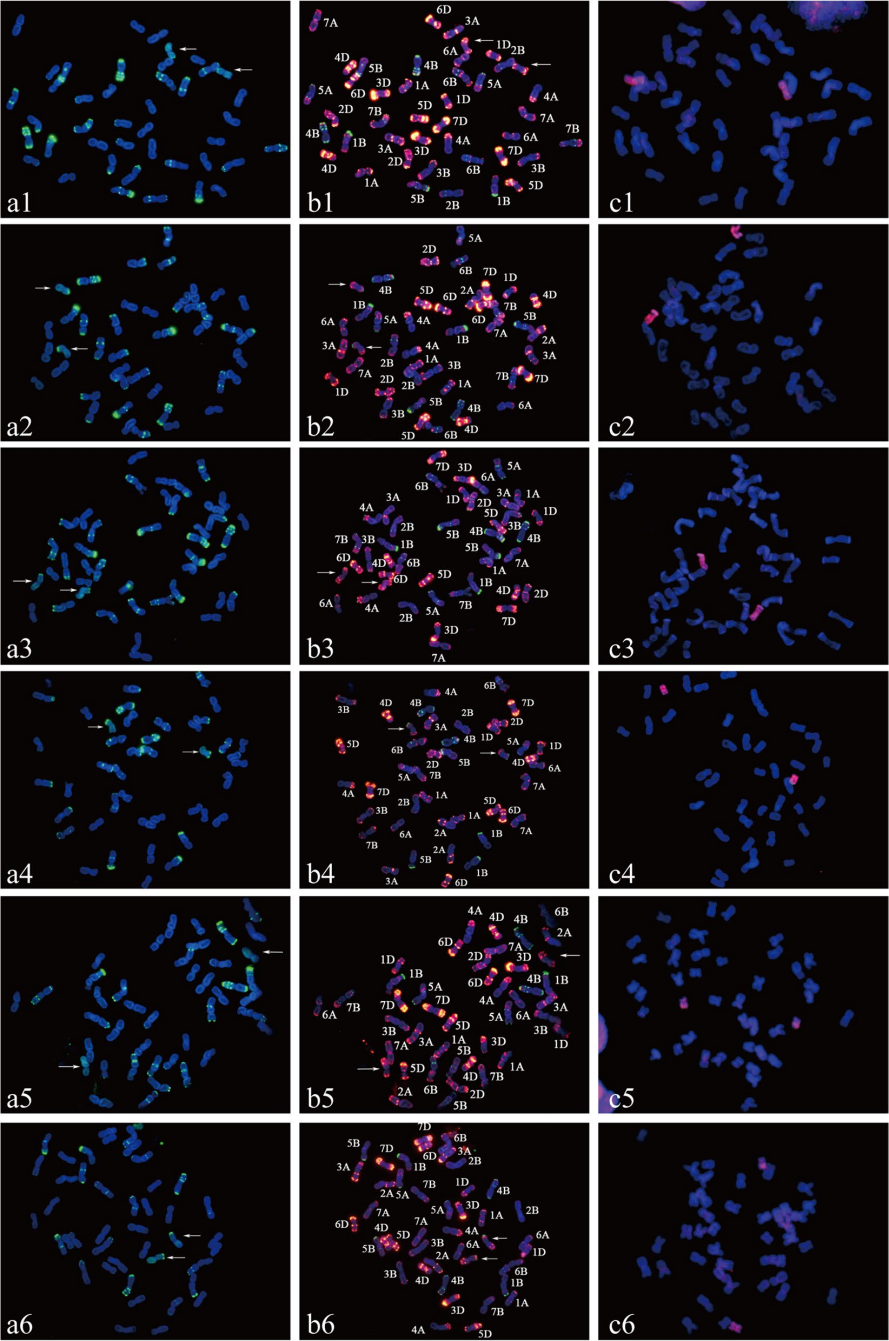
In situ hybridization patterns of the six alien substitution lines. **a **GISH patterns of ES-9 (a1) and ES-10 (a2): *Th. ponticum* genomic DNA (green) as probe and CS genomic DNA as a blocker; GISH patterns of ES-23 (a3), ES-24 (a4), ES-25 (a5), and ES-26 (a6): *Th. intermedium* genomic DNA (green) as probe and CS genomic DNA as a blocker. **b** FISH patterns of ES-9 (b1), ES-10 (b2), ES-23 (b3), ES-24 (b4), ES-25 (b5), and ES-26 (b6): Oligo-pSc119.2 (green) and Oligo-pTa535 (red) as probes.** c** Mc-GISH patterns of ES-9 (c1) and ES-10 (c2): *Th. bessarabicum* (J) genomic DNA (green) and tetraploid *P. spicata* (St) genomic DNA (red) as probes, CS genomic DNA as a blocker; ES-23 (c3), ES-24 (c4), ES-25 (c5), and ES-26 (c6): *Th. bessarabicum* (J) genomic DNA (green) and diploid *P. spicata* (St) genomic DNA (red) as probes, CS genomic DNA as a blocker. The arrows indicate the alien chromosomes of the six substitution lines

Alien chromosomes derived from *Th. ponticum* or *Th. intermedium* were traced using GISH analysis of somatic cells. All six alien lines, ES-9, ES-10, ES-23, ES-24, ES-25, and ES-26 contained 42 chromosomes (Fig. 1 and Fig S1). ES-9 and ES-10 both carried two *Th. ponticum* chromosomes with a bright-green hybridization signal based on using the *Th. ponticum* genome DNA as a probe (Fig. 1, a1 and a2). Whereas ES-23 (Fig. 1, a3), ES-24 (Fig. 1, a4), ES-25 (Fig. 1, a5), and ES-26 (Fig. 1, a6), carried two *Th. intermedium* chromosomes with a bright-green hybridization signal, based on the GISH probe of *Th. intermedium*. Therefore, ES-9 and ES-10 were wheat–*Th. ponticum* DSLs, and ES-23, ES-24, ES-25, and ES-26 were wheat–*Th. intermedium* DSLs.

Two oligonucleotide probes of pTa535 and pSc119.2 were combined for sequential FISH–GISH to simultaneously examine the elimination of wheat chromosomes in the six substitution lines. Comparisons of the FISH results between substitution lines and the corresponding parent lines, Abbondanza, Zhong4, and Xiaoyan784, were conducted. Chromosome 2A was eliminated in ES-9 and substituted by one pair of *Th. ponticum* chromosomes with three specific signal bands, including the terminal pTa535 hybridization sites detected on short arms and long arms as well as an interstitial pTa535 signal on the long arms, which was different from the FISH patterns of other wheat chromosomes (Fig. 1, b1). ES-10 lost chromosome 3D and contained one pair of *Th. ponticum* chromosomes carrying terminal pSc119.2 hybridization sites on short arms with terminal pTa535 hybridization segments both on the long arms and short arms (Fig. 1, b2). Wheat chromosomes 2A, 2B, and 2D were eliminated in ES-23 (Fig. 1, b3), ES-25 (Fig. 1, b5), and ES-26 (Fig. 1, b6), respectively, and replaced by the same pair of *Th. intermedium* chromosomes with identical FISH patterns to that detected in ES-9. Moreover, the telomeric region of chromosome 5B carrying a bright-green fluorescence signal was eliminated in ES-25 compared with other related materials. For ES-24, chromosome 3D was substituted by a pair of *Th. intermedium* chromosomes with the FISH patterns almost consistent with the alien chromosomes detected in ES-10 (Fig. 1, b4).

According to the multicolor GISH (mc-GISH) results, each of the six derived lines contained two alien chromosomes carrying a bright-red fluorescence signal originating from the *P. spicata* (St) genome DNA (Fig. 1, c1-c6). Combined with the sequential FISH-GISH analyses results, ES-9 and ES-10 carried two different pairs of St chromosomes derived from *Th. ponticum*. ES-23, ES-25, and ES-26 contained the same pair of St chromosomes from *Th. intermedium* which was different from the alien chromosomes of ES-24.

### Wheat 15 K SNP array analysis of the six substitution lines

**Fig. 2:**
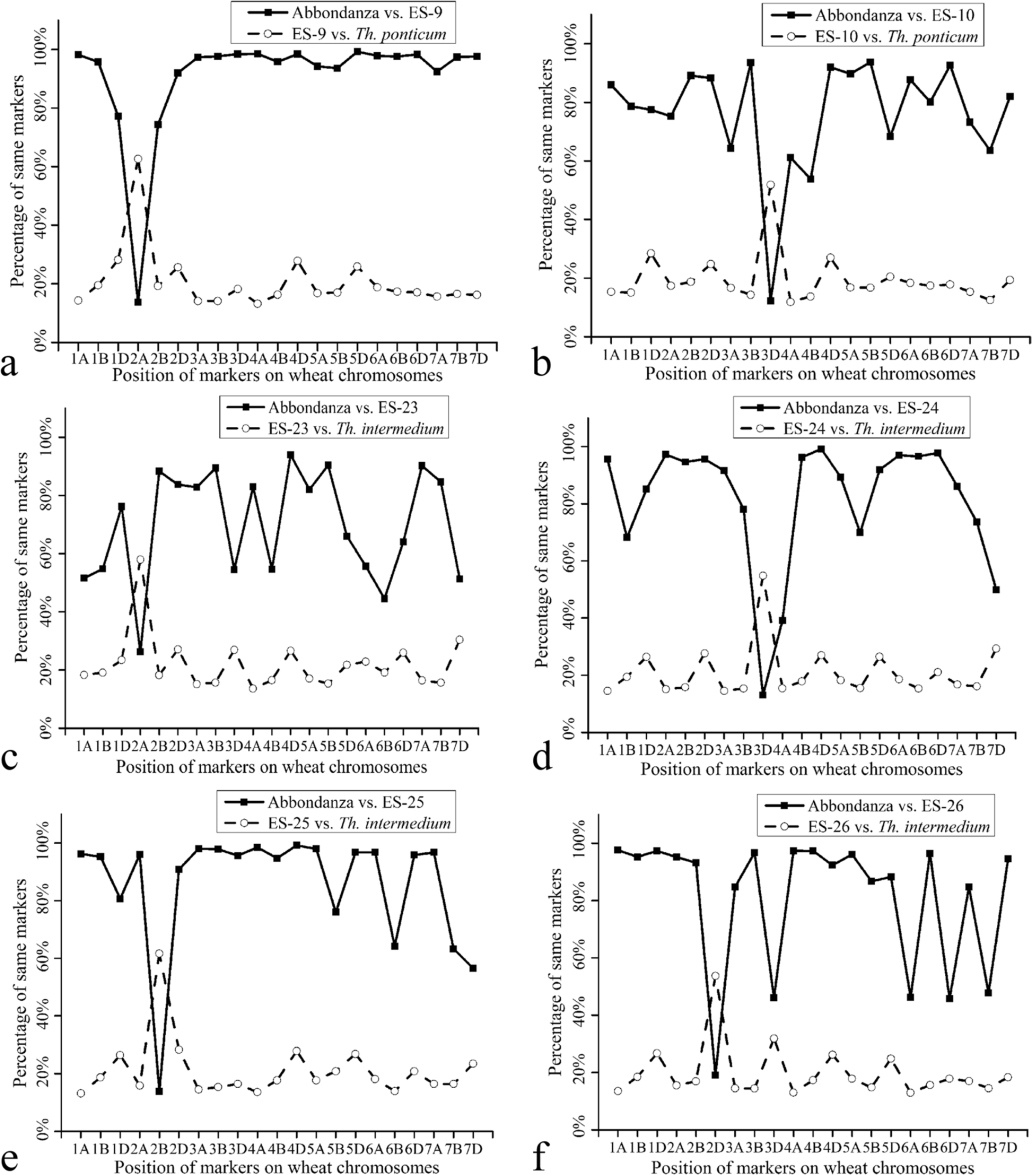
Wheat 15 K SNP array analysis of the six alien substitution lines. **a** Wheat 15 K SNP array analysis of ES-9. Obvious crossing points were detected in terms of the position of chromosomes 2A. **b** Wheat 15 K SNP array analysis of ES-10. Obvious crossing points were detected in terms of the position of chromosomes 3D. **c** Wheat 15 K SNP array analysis of ES-23. Obvious crossing points were detected in terms of the position of chromosomes 2A. **d** Wheat 15 K SNP array analysis of ES-24. Obvious crossing points were detected in terms of the position of chromosomes 3D. **e** Wheat 15 K SNP array analysis of ES-25. Obvious crossing points were detected in terms of the position of chromosomes 2B. **f** Wheat 15 K SNP array analysis of ES-26. Obvious crossing points were detected in terms of the position of chromosomes 2D

The chromosomal compositions of the six substitution lines were further determined based on genotype data using a wheat 15 K SNP array (Table S1). Generally, compared with *Th. ponticum* or *Th. intermedium*, the number of common SNP sequences detected between the substitution lines and Abbondanza was much higher. However, an obvious point of intersection was found in each of the substitution lines (Fig. 2a-f). For ES-9 (Fig. 2a), the intersection point was distinct in chromosome 2A, and ES-9 had mostly the same SNP marker loci as *Th. ponticum* but few SNP marker loci as Abbondanza. These results suggested that chromosome 2A in ES-9 was replaced by the alien chromosomes of *Th. ponticum*, which was consistent with the FISH result. In ES-10 (Fig. 2b), the intersection point was detected in chromosome 3D, and ES-10 had mostly the same SNP marker loci as *Th. ponticum* but shared few SNP marker loci with Abbondanza, suggesting that chromosome 3D of ES-10 was substituted by the pair of *Th. ponticum* chromosomes.

In terms of the wheat–*Th. intermedium* alien lines, the intersection point was detected in chromosome 3D of ES-24, and ES-24 had mostly the same SNP marker loci as *Th. intermedium*. Thus, chromosome 3D of ES-24 was replaced by the pair of *Th. intermedium* chromosomes (Fig. 2d). The intersection points of ES-23, ES-25, and ES-26 were respectively identified in chromosomes 2A (Fig. 2c), 2B (Fig. 2e), and 2D (Fig. 2f). Combined with the FISH results, it was revealed that chromosome 2A in ES-23, chromosome 2B in ES-25, and chromosome 2D in ES-26 were substituted by the same pair of *Th. intermedium* chromosomes.

### PLUG marker analysis of the six substitution lines

**Fig. 3:**
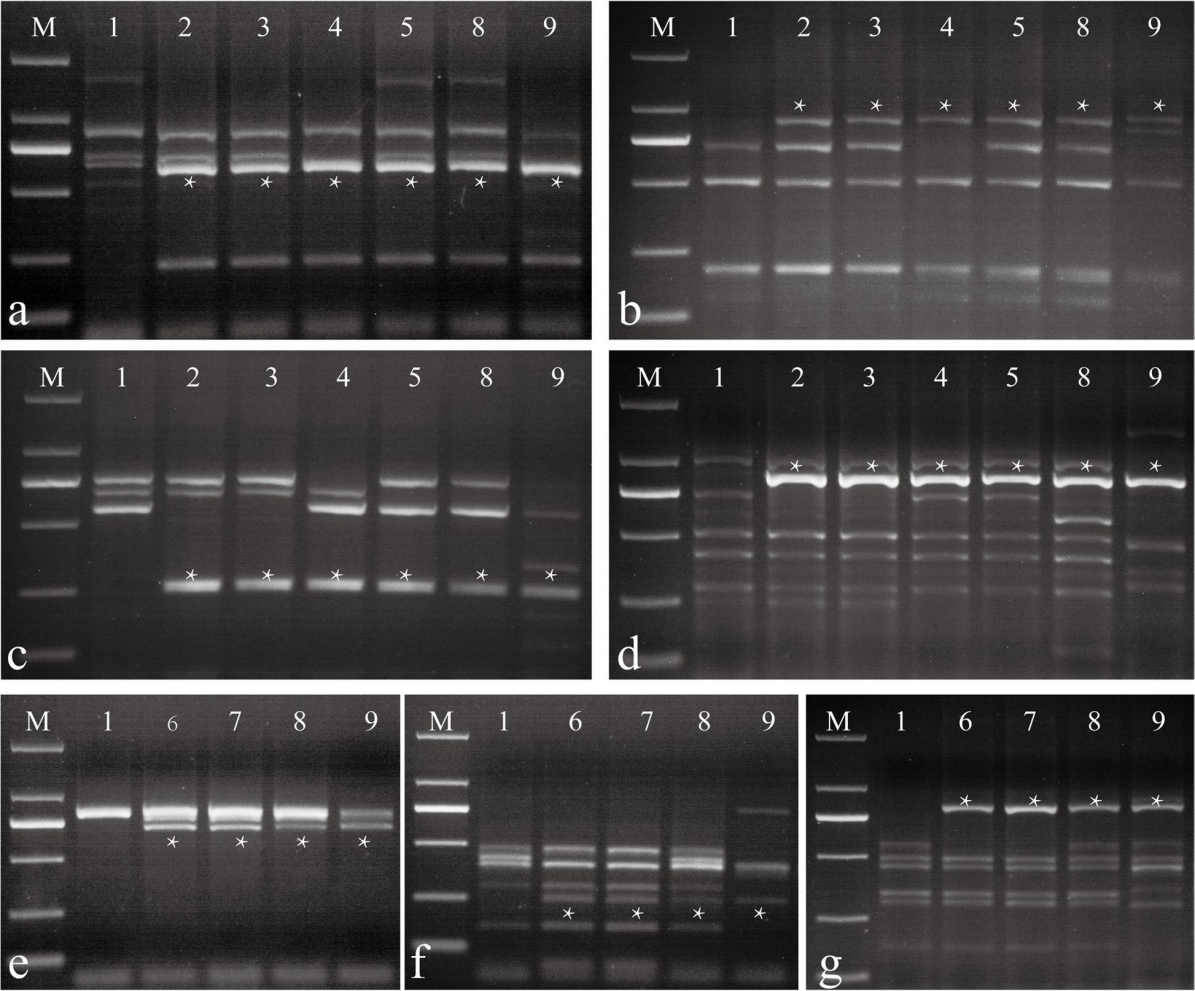
PLUG markers analysis of Abbondanza, the six alien substitution lines,* Th. intermedium*, and *Th. ponticum*. **a***TNAC1142-Hae*III; **b***TNAC1142-Taq*I; **c***TNAC1132-Taq*I; **d***TNAC1140-Taq*I; **e***TNAC1326-Hae*III; **f***TNAC1326-Taq*I; **g***TNAC1359-Taq*I. Lane M: DL2000; lane 1: Abbondanza; lane 2: ES-9; lane 3: ES-23; lane 4: ES-25; lane 5: ES-26; lane 6: ES-10; lane 7: ES-24; lane 8: *Th. intermedium*; lane 9: *Th. ponticum*. The * indicates specific band of* Th. ponticum* and *Th. intermedium*

The 135 PLUG markers were screened to validate the homoeologous groups for the alien chromosomes. Four PLUG markers (*TNAC1142-Hae*III, *TNAC1142-Taq*I, *TNAC1132-Taq*I, and *TNAC1140-Taq*I) were mapped to the second homoeologous group in ES-9, ES-23, ES-25, and ES-26 (Fig. 3a-d, Table S2 and Fig. S2). Three pairs of primers (*TNAC1326-Hae*III, *TNAC1326-Taq*I, and *TNAC1359-Taq*I) were distributed in the third homoeologous group in ES-10 and ES-24 (Fig. 3e-g, Table S2 and Fig. S2). Combined with the mc-GISH results of each substitution line, the 2St-chromosome-specific bands could be amplified in ES-9, ES-23, ES-24, ES-25, ES-26, *Th. intermedium*, and *Th. ponticum*. 3St-chromosome-specific bands were identified in ES-10, ES-24, *Th. intermedium*, and *Th. ponticum*. The above polymorphic bands could not be amplified in Abbondanza.

**Fig. 4:**
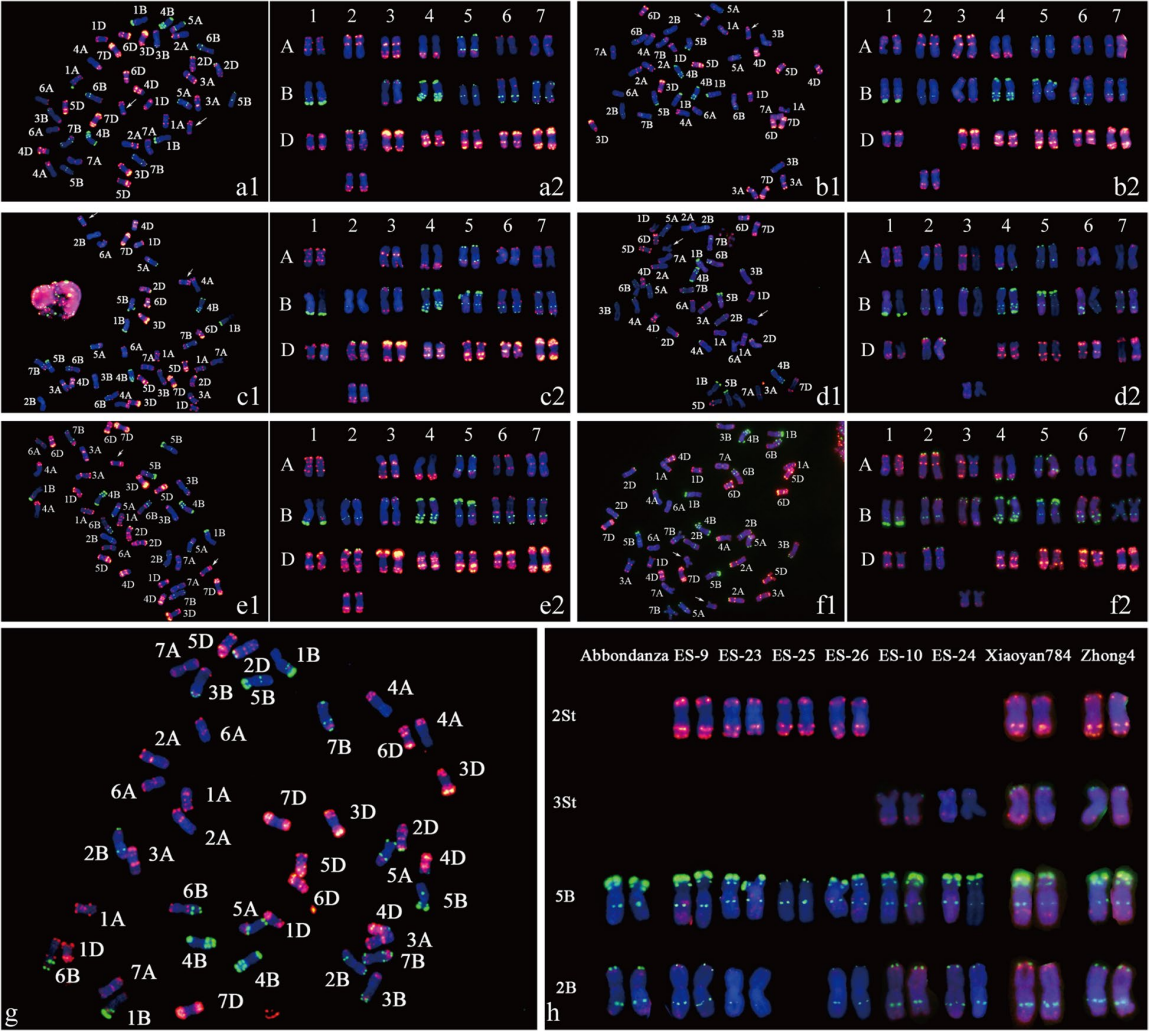
Karyotypes of the six alien substitution lines with genomic composition variations. **a** Karyotype analysis of ES-25. Wheat chromosomes 2B were replaced by *Thinopyrum intermedium* chromosomes 2St. **b** Karyotype analysis of ES-26. Wheat chromosomes 2D were replaced by *Th. intermedium* chromosomes 2St. **c** Karyotype analysis of ES-23. Wheat chromosomes 2A were replaced by *Th. ponticum* chromosomes 2St. **d** Karyotype analysis of ES-24. Wheat chromosomes 3D were replaced by *Th. intermedium* chromosomes 3St. **e** Karyotype analysis of ES-9. Wheat chromosomes 2A were replaced by *Th. intermedium* chromosomes 2St. **f** Karyotype analysis of ES-10. Wheat chromosomes 3D were replaced by *Th. intermedium* chromosomes 3St. **g** FISH analysis of Abbondanza. **h** FISH pattern comparisons of chromosomes 2St, chromosomes 3St, chromosomes 5B, and chromosomes 2B between the six alien substitution lines and their parent lines Abbondanza, Xiaoyan784, and Zhong4. The telomeric region of chromosome 5BS carrying a bright-green fluorescence signal was eliminated in ES-25. Chromosomes 2B are metacentric in all the above-mentioned materials except ES-25

Based on chromosomal composition analysis (Fig. 4 and Fig. S3), the genome composition of ES-25 (Fig. 4a) was 14A + 12B + 14D + 2(2St), and ES-26 (Fig. 4b) was 14A + 14B + 12D + 2(2St). ES-23 and ES-9 had the same genome composition of 12A + 14B + 14D + 2(2St), while ES-24 and ES-10 had the same genome composition of 14A + 14B + 12D + 2(3St). Remarkably, the alien chromosomes contained in ES-23 (Fig. 4c) and ES-24 (Fig. 4d) were derived from *Th. intermedium*, while the alien chromosomes carried by ES-9 (Fig. 4e) and ES-10 (Fig. 4f) were derived from *Th. ponticum*. Although the alien chromosomes belonging to the same homoeologous groups were derived from two different donors, identical FISH karyotypes of the alien chromosomes were detected between ES-23 and ES-9 (2St), as well as ES-24 and ES-10 (3St). Additionally, FISH result of Abbondanza was shown in Fig. 4g, and the FISH pattern comparisons of above materials were shown in Fig. 4h. Notably, ES-10 has a similar chromosome composition to our previously reported ES-12 [33], but the common wheat background is different in the FISH karyotype (as shown in Figs. S3 and S4).

### Evaluation of agricultural performance and resistance to stripe rust of the six substitution lines

**Fig. 5:**
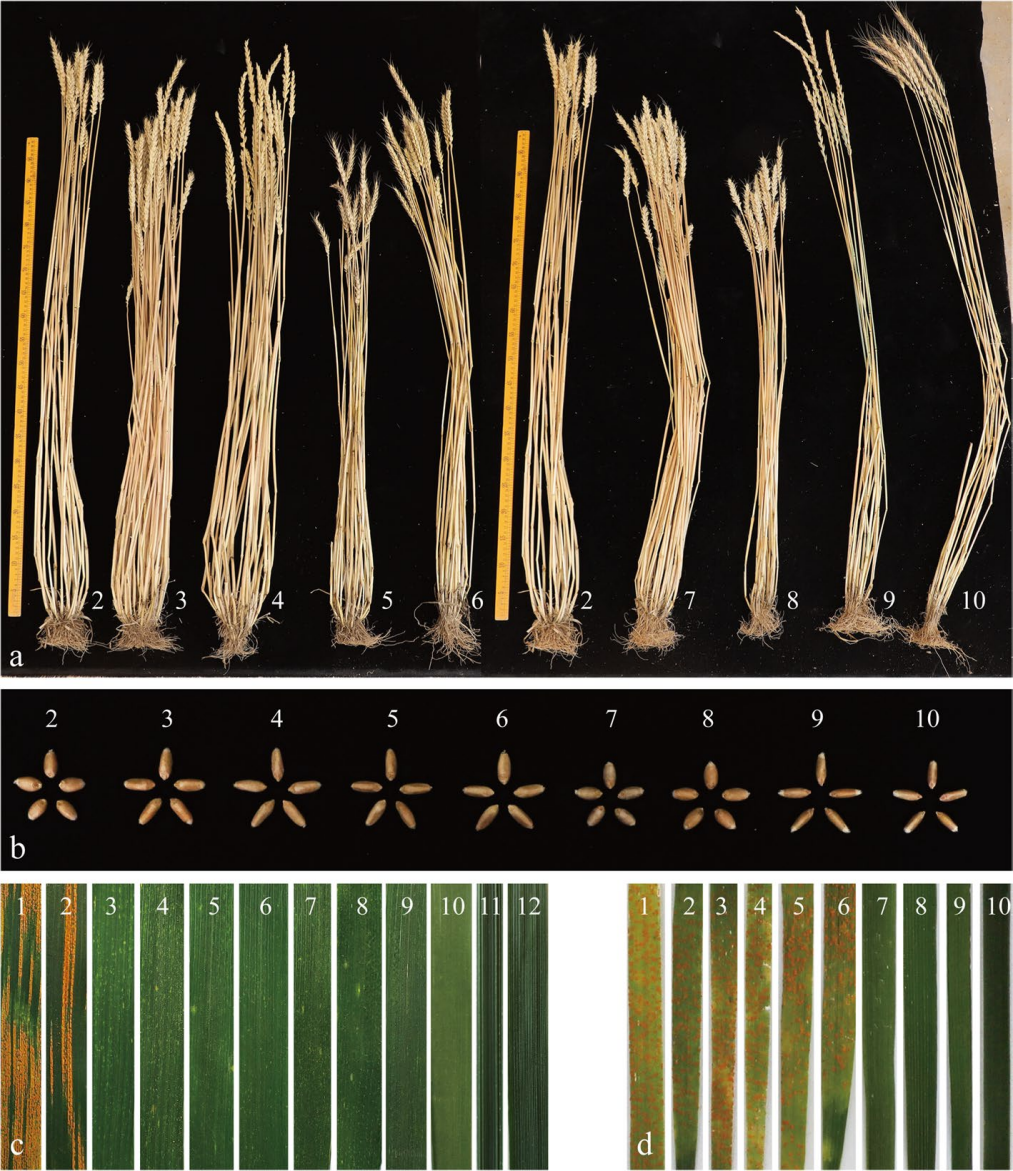
Evaluation of agronomic traits and stripe rust resistance. **a** Adult plants; **b** seeds; **c** symptoms in response to inoculation with the mixture of *Pst* races at the adult stage; **d** seedling stage reactions to *Pst* race CYR32. (1) Huixianhong; (2) Abbondanza; (3) ES-9; (4) ES-23; (5) ES-25; (6) ES-26; (7) ES-10; (8) ES-24; (9) Xiaoyan784; (10) Zhong4; (11) *Th. ponticum*; (12) *Th. intermedium*

The agronomic traits of the six substitution lines as well as their parents Abbondanza and Xiaoyan784 (Table 1; Fig. 5) or Zhong4 (Table 2; Fig. 5) were compared. On average, the tiller number of ES-9 was higher and the spikes were longer than Abbondanza. In terms of the substitution lines derived from Zhong4, both ES-23 and ES-26 showed many more tillers, and the number of spikelets per spike in ES-26 was higher than Abbondanza and Zhong4. Surprisingly, the average thousand-kernel weights of the alien lines containing chromosome 2St (ES-9, ES-23, ES-25, and ES-26) were more than 43 g. This indicated that chromosome 2St increased thousand-kernel weight whether originating from *Th. ponticum* or *Th. intermedium*.

**Table 1:** Agronomic traits of the alien substitution lines ES-9, ES-10, as well as their parents (Abbondanza, Xiaoyan784)

Materials	Plant height (cm)	Tillers	Spike length (cm)	Spikelets/ spike	Seedss/ spikelet	Thousand Kenel Weight (g)	Awnedness
Xiaoyan784	112 ± 6a	11 ± 3b	21 ± 1.5a	25 ± 2a	6 ± 1a	31 ± 0.5c	awnless
ES-9	105 ± 5a	25 ± 4a	13.9 ± 1b	24 ± 1ab	4 ± 1b	43 ± 1a	awnless
ES-10	90 ± 5b	18 ± 4ab	14 ± 1.5b	20 ± 1c	5 ± 1ab	36 ± 1b	awnless
Abbondanza	107 ± 6a	14 ± 3b	14.4 ± 1b	22 ± 1b	4 ± 1b	42 ± 2a	awnless

Different letters a, b and c indicate significant differences between ES-9, ES-10 and its wheat parent (*P* < 0.05)

**Table 2:** Agronomic traits of the substitution lines ES-23, ES-24, ES-25, ES-26, as well as their parents (Abbondanza and Zhong4)

Materials	Plant height (cm)	Tillers	Spike length (cm)	Spikelets/ spike	Seeds/ spikelet	Thousand Kenel Weight (g)	Awnedness
Zhong4	127 ± 4a	12 ± 3b	16 ± 1.5a	23 ± 2ab	5 ± 1a	31 ± 0.5c	Long awn
ES-23	114 ± 5b	23 ± 4a	17 ± 1.5a	21 ± 2bc	4.4 ± 1ab	43 ± 1a	awnless
ES-24	87 ± 6c	17 ± 4b	11.6 ± 1c	21 ± 1c	4.4 ± 1ab	37 ± 2b	Short awn
ES-25	93 ± 5c	15 ± 4b	13 ± 1.5b	23 ± 1b	3.7 ± 1b	45 ± 1.5a	Short awn
ES-26	113 ± 5b	27 ± 4a	14 ± 1.5b	25 ± 1a	4.4 ± 1ab	43 ± 1.5a	Short awn
Abbondanza	107 ± 6b	14 ± 3b	14.4 ± 1b	22 ± 1b	4 ± 1b	42 ± 2ab	awnless

Different letters a, b and c indicate significant differences between ES-23, ES-24, ES-25, ES-26 and its wheat parent (*P* < 0.05)

At the adult stage, a stripe rust reaction test of the six substitution lines was carried out with the susceptible control (HXH). Sequentially, the infection type (IT) scores of the six substitution lines, Abbondanza, Xiaoyan784, Zhong4, as well as *Th. ponticum* and *Th. intermedium* were recorded under field conditions. The IT scores of the above-mentioned materials were as follows: *Th. ponticum*, IT = 0; *Th. intermedium*, IT = 0; Xiaoyan784, IT = 0; Zhong4, IT = 0; ES-9, IT = 1; ES-10, IT = 0; ES-23, IT = 1; ES-24, IT = 0; ES-25, IT = 1; ES-26, IT = 1; Abbondanza, IT = 3; HXH, IT = 4 (Fig. 5c). Furthermore, stripe rust infection of the seedling stage was conducted in the greenhouse, and the IT scores were recorded at 24 days post-inoculation (Fig. 5d). With an IT score of 0, Zhong4 and Xiaoyan784 were immune to the disease. Additionally, ES-10 and ES-24 were nearly immune (IT scores of 1). In contrast, Abbondanza, ES-9, ES-23, ES-25, and ES-26 were susceptible (IT scores of 3). The results suggested that chromosome 2St originating from *Th. ponticum* and *Th. intermedium* both conferred resistance to stripe rust at the adult stage, while chromosome 3St of *Th. ponticum* and *Th. intermedium* both carried remarkable resistance at all stages.

### Meiotic chromosome pairing analysis of F_1_ hybrids

Crosses were made between the alien lines with the same genome compositions. Fifteen F_1_ plants were obtained from the cross between ES-9 and ES-23, and 11 F_1_ plants were obtained from the cross between ES-10 and ES-24. Meiotic chromosome pairing analysis of the F_1_ hybrids was conducted to further validate the related genome constitution (Table 3). Mostly, the alien chromosomes derived from *Th. intermedium* and *Th. ponticum* but belonging to the same homoeologous group (2/3) could form a bivalent at metaphase I, and no trivalent or quadrivalent was observed at meiosis anaphase I. These results further revealed the close homoeologous relationship between the alien chromosomes derived from the two different donors.

**Table 3:** Chromosome pairing in the meiotic and meiotic phases for the hybrid F_1_ individuals

**Material**	**No. of cells**	**Chromosome configuration**					
		**Univalent**	**Bivalent**			**Trivalent**	**Quadrivalent**
			**Rod**	**Ring**	**Total**		
**ES-9 × ES-23**	**144**	**0.47(0–2)**	**2.69(1–4)**	**17.93(17–21)**	**20.62(20–21)**	**0**	**0**
**ES-10 × ES-24**	**135**	**0.39(0–2)**	**2.61(1–4)**	**18.19(17–21)**	**20.8(20–21)**	**0**	**0**

### Comparisons of genomic polymorphism analyses and St-chromosomes-specific molecular markers development

After high-throughput sequencing, a SLAF library was constructed with the sequencing details (Table S3). A total of 1,055,234 (ES-9), 938,861 (ES-10), 524,288 (ES-23), 1,026,271 (ES-24), 974,634 (Abbondanza), 572,791 (*Th. intermedium*), and 513,056 (*Th. ponticum*) SLAFs were obtained. By bioinformatics analysis, 3,203 (ES-9), 4,455 (ES-23), 2,775 (ES-10), and 3,148 (ES-24) specific sequences were selected for further sequence alignments. There were 78 out of 263 sequences from ES-24 with homology of more than 90% with ES-10 (78/153). In addition, 114 out of 221 sequences from ES-23 were more than 90% homologous with ES-9 (114/177). To some degree, these results revealed the possible genomic similarity between the alien chromosomes of *Th. intermedium* and *Th. ponticum*.

**Fig. 6:**
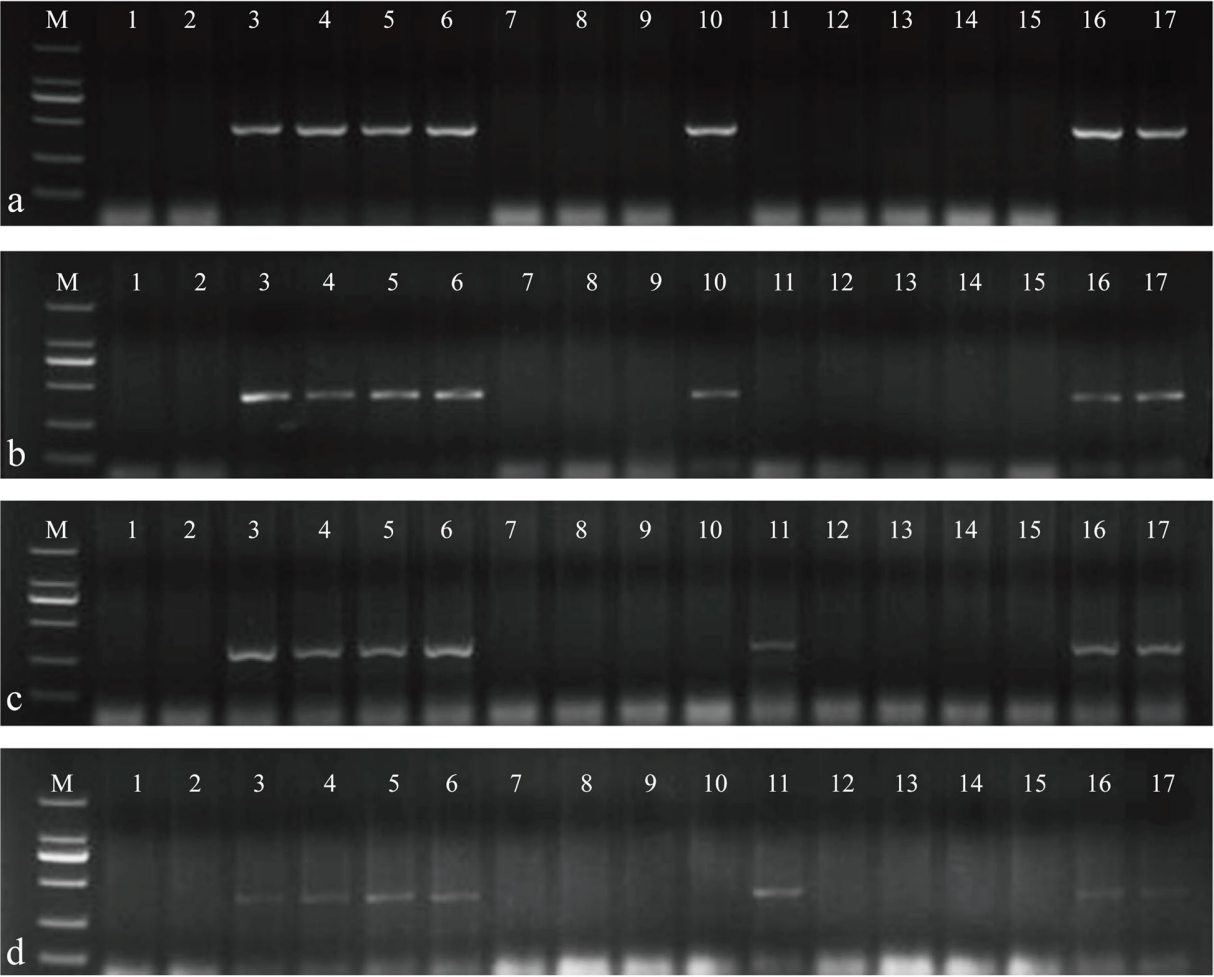
Specific amplification markers of chromosome 2St (**a** and **b**) and chromosome 3St (**c** and **d**). **a** PTH-005; **b** PTH-013; **c** PTH-113; **d** PTH-135. Lane M: DL2000; lane 1: Chinese Spring; lane 2: Abbondanza; lane 3: *Th. ponticum*; lane 4: *Th. intermedium*; lane 5: tetraploid *P. spicata*; lane 6: diploid *P. spicata*; lane 7: *Th. bessarabicum*; lane 8: *Th. elongatum*; lane 9–15: wheat–*Th. intermedium* disomic addition lines (DALs), DA1St, DA2St; DA3St; DA4St; DA5St; DA6St; DA7St; lane 16: ES-9 (**a** and **b**), ES-10 (**c** and **d**); lane 17: ES-23 (**a** and **b**), ES-24 (**c** and **d**)

According to the above sequence alignment results, 110 SLAFs from ES-23 regarded as 2St chromosome-specific fragments and 73 SLAFs from ES-24 regarded as 3St chromosome-specific fragments were selected. Subsequently, 183 pairs of primers were designed to amplify fragments from CS, Abbondanza, Zhong4, Xiaoyan784, ES-9, ES-23, ES-10, and ES-24. Specificity of the primers was further confirmed by analysis of *Th. ponticum*, *Th. intermedium*, tetraploid *P. spicata*, diploid *P. spicata*, *Th. bessarabicum*, *Th. elongatum*, and the wheat–*Th. intermedium* 1-7St addition line. In total, two 2St-chromosome-specific molecular markers, PTH-005 and PTH-013, and two 3St-chromosome-specific molecular markers, PTH-113 and PTH-135, were developed (Fig. 6, Table 4 and Fig. S5).

**Table 4:** Specific amplification markers of chromosome 2St and chromosome 3St

Specific primers	Primers (5’-3’)	Amplified chromosomes	Annealing temperatures
**PTH-005**	F: TCCTCAACTGGAAACAAAGGA	2St	56
	R: TTGGGAGTGAGTGTAGTTCAC		
**PTH-013**	F: AGCCCTCCGGAAAGAATGAA	2St	62
	R: CCGCTCAAACAATCGCTACC		
**PTH-113**	F: AACAGGGTCAACGGGTTTGA	3St	60
	R: TTGGTGCAGAAACAATGCGG		
**PTH-135**	F: TGCCTCTAACACATGCATGT	3St	60
	R: TCCAGTAGGTCTTGGCTCCA

### Utility of the 3St-chromosome-specific markers in a BC_1_F_2_ population

**Fig. 7:**
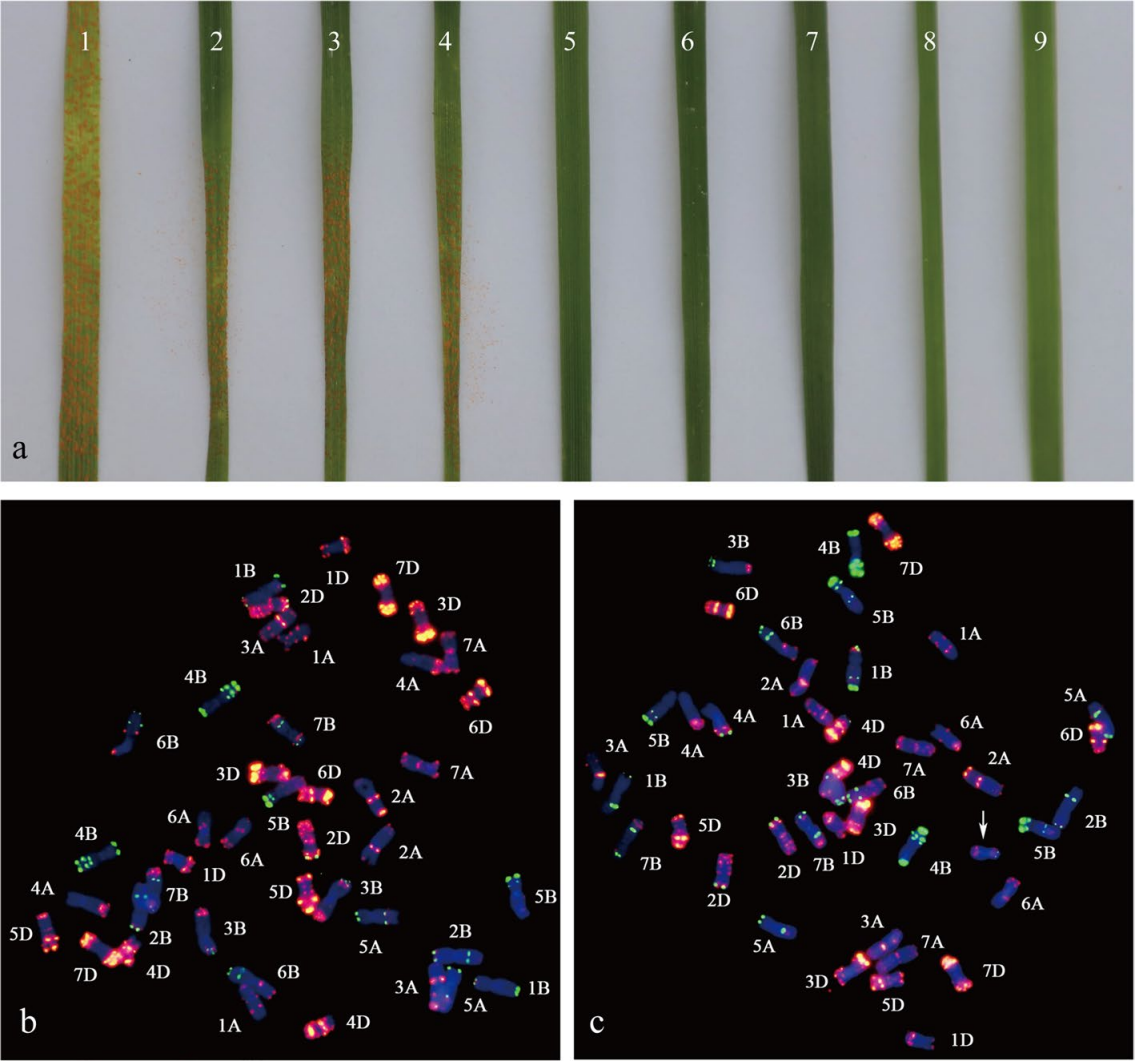
Stripe rust resistance evaluation and FISH analysis in BC_1_F_2_ individuals of ES-24 and Huixianhong (HXH). **a** Reactions to inoculation with the *Pst* race CYR32 of the BC_1_F_2_ individuals at the seedling stage; **b** FISH patterns of susceptible BC_1_F_2_ individuals; **c** FISH patterns of resistant BC_1_F_2_ individuals. (1) HXH; (2)-(4) susceptible BC_1_F_2_ individuals; (5)-(7) resistant BC_1_F_2_ individuals; (8) Zhong4; (9) ES-24. The arrow indicates the chromosome 3St of *Th. intermedium*

In order to validate that the stripe rust resistance gene(s) at all stages were carried by chromosome 3St, 60 BC_1_F_2_ individuals of ES-24 and HXH were used for a genetic analysis. The evaluation of stripe rust resistance at the seedling stage revealed that Zhong4, ES-24, and the 33 F_2_ individuals were highly resistant to *Pst* race CYR32 (Fig. 7a). Subsequently, ten resistant F_2_ individuals as well as ten susceptible individuals were randomly selected for FISH analysis. Compared with the FISH karyotype of ES-24, susceptible individuals had undetectable FISH patterns of chromosome 3St (Fig. 7b), while chromosome 3St was detected in all the resistant individuals (Fig. 7c). These results demonstrated that the novel stripe rust resistant gene(s) originated from the alien chromosome 3St of *Th. intermedium*.

**Fig. 8:**
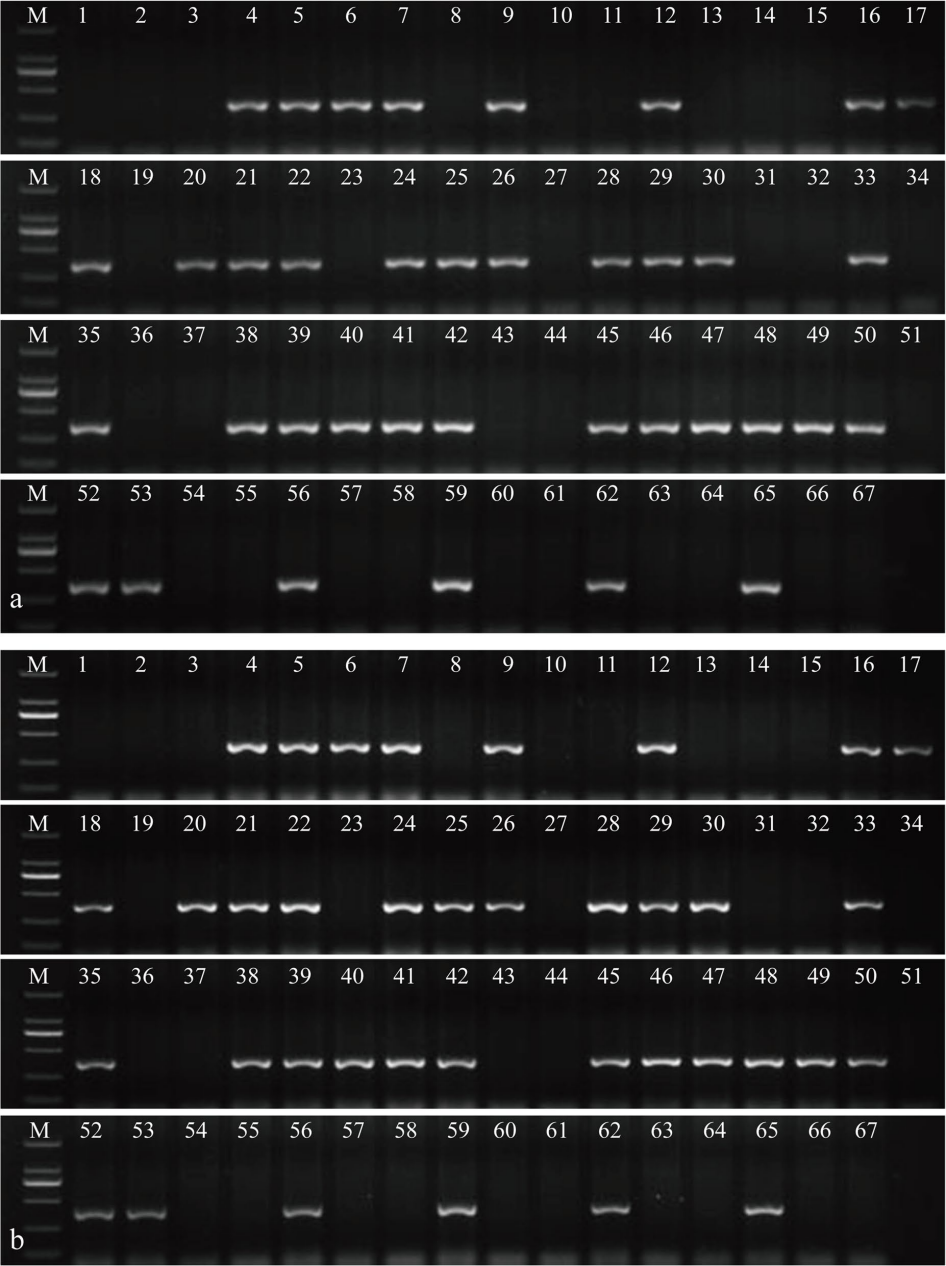
Utility of newly developed 3St-chromosome-specific markers in 60 BC_1_F_2_ individuals of ES-24 and HXH. **a** PTH-113; **b** PTH-135. Lane M: DL2000; lane 1: Chinese Spring; lane 2: Abbondanza; lane 3: HXH; lane 4: Xiaoyan784 (wheat–*Th. ponticum* partial amphiploid with stripe rust); lane 5: Zhong4 (wheat–*Th. intermedium* partial amphiploid with stripe rust); lane 6: ES-10; lane 7: ES-24; lane 8–67: 60 BC_1_F_2_ individuals

Furthermore, the specificity of PTH-113 and PTH-135 was confirmed by PCR analyses of the 60 BC_1_F_2_ individuals (Fig. 8 and Fig. S6). Combined with the result of seedling stage stripe rust resistance evaluation, Xiaoyan784, Zhong4, ES-9, ES-24, and the 33 BC_1_F_2_ plants conferring strong resistance to *Pst* race CYR32 also carried 3St chromosome-specific markers. In contrast, the other 27 BC_1_F_2_ plants, the parental line Abbondanza, and susceptible control HXH, without specific amplification, were seriously susceptible to *Pst* race CYR32. Hence, the new developed chromosome-specific molecular markers could be used to rapidly trace the alien chromosome 3St in a common wheat background.

## Discussion

Chromosome manipulation for distant hybridization has been widely utilized for wheat improvement programs, especially for breeding novel disease-resistant wheat lines. Over the past few decades, numerous disease-resistance genes carried by wild species related to wheat have been successfully transferred to a common wheat background by developing introgression lines [37–42]. DSLs containing one pair of alien chromosomes with desirable resistance genes are vital bridge materials for small segments of introgression [43–45], and serve as valuable germplasm resources for wheat disease-resistance breeding. In the current study, six stable wheat–*Thinopyrum* derived lines were developed using a nullisomic backcross method. Molecular cytogenetic analysis confirmed that ES-23 (DS2St (2A)), ES-24 (DS3St (3D)), ES-25 (DS2St (2B)) and ES-26 (DS2St (2D)) are wheat–*Th. intermedium* DSLs, while ES-9 (DS2St (2A)) and ES-10 (DS3St (3D)) are wheat–*Th. ponticum* DSLs. The four alien lines, ES-9, ES-23, ES-25 and ES-26 carrying chromosome 2St conferred higher thousand-kernel weight and stripe rust resistance at adult stages, while ES-10 and ES-24 both containing chromosome 3St were highly resistant to stripe rust at all stages. Therefore, all six substitution lines may serve as novel resistant germplasms for wheat breeding.

One of the most commonly used techniques, FISH analysis, is generally used with GISH to discriminate alien chromosomes [33, 46] and to detect genomic changes in specific regions [47–49]. In this study, after characterization by sequential FISH–GISH and mc-GISH analysis, specific karyotype patterns of chromosomes 2St and 3St derived from *Th. intermedium* and *Th. ponticum* were elucidated, which is useful for rapidly identifying the alien chromosomes in germplasm materials. Furthermore, chromosomal structure variation was observed in ES-25 when distant hybridization was detected by FISH. Compared with the parental lines, Abbondanza and Zhong4, the telomere with the subtelomeric region of chromosome 5BS carrying a blight pSc119.2 hybridization signal was eliminated in ES-25, resulting in a similar FISH pattern to chromosome 2B of common wheat. Chromosome 2B is almost metacentric whereas chromosome 5B is fully submetacentric, so it was clear that chromosome 2B was replaced by chromosome 2St of *Th. intermedium* (Fig. 4h). Due to the dynamic and high frequency variation of subtelomeres in Triticeae species [50, 51], it is difficult to identify the possible function(s) of the deleted regions of chromosome 5BS. Because there were no severe effects on viability of ES-25, elimination of the subtelomeric region presumably contributed to genome diversity [52].

The genomic composition of *Th. ponticum* and *Th. intermedium* has been an interesting subject for many years [53, 54]. During the past several decades, it was determined that the set of St chromosomes contained in *Th. intermedium* were probably derived from *P. spicata* [55], whereas it was unclear whether the St genome is one of the sets of chromosomes of *Th. ponticum* [11, 56]. According to molecular cytogenetic identification results, ES-23 and ES-9 (group 1) contained the same genome composition of 12A + 14B + 14D + 2(2St), while ES-24 and ES-10 (group 2) had the same genome composition of 14A + 14B + 12D + 2(3St). Although the alien chromosomes were derived from two different donors, *Th. intermedium* and *Th. ponticum*, identical alien chromosome FISH patterns and similar specific agricultural performances were identified in each group of plant materials. This implies that St chromosomes were included in *Th. ponticum*, and could be stably inherited with desirable genes. Furthermore, combined with the close homoeologous relationships between the alien chromosomes analyzed by meiotic chromosome pairing and genomic polymorphism, *P. spicata* was identified to represent the complete set of St chromosomes that function directly during the speciation of *Th. ponticum*. However, further analyses are needed to determine the effects of the recombination events that occurred between diverse genomes through the allopolyploidization. In summary, based on the specific SLAFs obtained in this study, it was feasible for us to develop transferable St-chromosome-specific molecular markers from *Th. intermedium* to *Th. ponticum*.

Although FISH–GISH analysis has been widely utilized to precisely characterize wheat–*Th. intermedium* lines for several decades, it is very time-consuming. Specific molecular markers can rapidly trace alien chromosomes or even small segment introgression with advantageous traits for wheat improvement breeding programs. However, the complete *Th. intermedium* genome has not been sequenced, so only a few chromosome-specific markers are available [57–59]. With the development of sequencing technology, the first consensus genetic map of *Th. intermedium* was developed by genotyping-by-sequencing [60]. Subsequently, 635 [9] and 745 [61] unique *Th. intermedium* SNP markers have been successfully developed, including 225 St-chromosome-specific markers, with 27 2St-chromosome-specific markers and 25 3St-chromosome-specific markers. Due to the much more complex genomic composition of *Th. ponticum*, molecular marker development work is mainly focused on genome E [56, 62, 63], especially following publication of the complete genome of *Th. elongatum* [64].

In the present study, the wheat–*Th. intermedium* DSLs, ES-23(DS2St (2A)) and ES-24(DS3St (3D)) were sequenced by SLAF-seq for further St-chromosome-specific marker development. Two 2St-chromosome-specific molecular markers, PTH-005 and PTH-013, as well as two 3St-chromosome-specific molecular markers, PTH-113 and PTH-135, were obtained. FISH analysis of the BC_1_F_2_ population of ES-24 and HXH combined with a stripe rust resistance test (Fig. 7) confirmed that the stripe rust resistance gene(s) was (were) derived from chromosome 3St of *Th. intermedium*. The utility of PTH-113 and PTH-135 amplification in the BC_1_F_2_ individuals indicated that the St-chromosome-specific molecular markers can serve as useful tools for tracing chromosome 3St of *Th. intermedium* in a common wheat background. In addition, according to the close genetic relationship between the alien chromosomes of *Th. ponticum* and *Th. intermedium* analyzed in this study, the four St-chromosome-specific markers could be simultaneously amplified in *Th. ponticum*, tetraploid *P. spicata*, *Th. intermedium*, and diploid *P. spicata*, as well as the corresponding substitution lines, ES-9, ES-23, ES-10, and ES-24. These results suggested that the four St-chromosome-specific markers could also be utilized to rapidly detect the St genome chromosomes of *Th. ponticum*. The remarkable stripe rust resistance of ES-24 and ES-10 thus probably originated from the same gene(s), but this needs to be validated in future genetic analyses.

## Conclusions

Four wheat–*Th. intermedium* and two wheat–*Th. ponticum* DSLs conferring stripe rust resistance were characterized and compared by molecular cytogenetic analysis, and can be used as bridging parents for transmission of valuable resistance genes. Furthermore, according to the related homoeologous relationships, two 2St-chromosome-specific and two 3St-chromsome-specific molecular markers were developed by SLAF-seq for rapidly detecting the alien chromosomes of *Th. intermedium* and *Th. ponticum* in a common wheat background.

## Materials and methods

### Plant materials

The plant materials included *Th. intermedium* (2*n* = 6*x* = 42, JJJ^s^J^s^StSt), *Th. ponticum* (2*n* = 10*x* = 70), diploid *P. spicata* (2*n* = 2*x* = 14, StSt), tetraploid *P. spicata* (2*n* = 4*x* = 28, StStStSt), *Th. bessarabicum* (2*n* = 2*x* = 14, JJ), *Th. elongatum* (2*n* = 2*x* = 14, EE), wheat cv. Chinese Spring (CS), the Abbondanza lines, ES-9, ES-10, ES-23, ES-24, ES-25, ES-26, Zhong4, and Xiaoyan784, as well as two wheat–*Th. intermedium* disomic addition lines (DALs), L4 (DA4St) and L7 (DA6St). Twenty-six F_1_ hybrids were obtained from two cross combinations, ES-9 × ES-23 (15 plants), and ES-10 × ES-24 (11 plants). The BC_1_F_2_ population comprising 60 individuals was derived from ES-24 and the wheat landrace Huixianhong (HXH). Five wheat–*Th. intermedium* DALs were developed via hybridization between Abbondanza nullisomic lines and Zhong4, including DA1St, DA2St, DA3St, DA5St, and DA7St (unpublished data). All the above-mentioned plant materials were preserved at the College of Agronomy, Northwest A&F University, China. HXH served as a susceptible control in the stripe rust resistance evaluation. The *Pst* race CYR32 was used for seedling stage of stripe rust resistance evaluation, and the CYR31 and CYR32 mixture was used for adult stage evaluation. All the *Pst* races were provided by the College of Plant Protection, Northwest A&F University, China.

### In situ hybridization

Chromosome spreads by the drop method [14] were used for in situ hybridization analyses. The protocols of genomic DNA extraction, sequential FISH–GISH, and mc-GISH were based on Wang et al. [33]. According to the nick translation method, total genomic DNA of *Th. bessarabicum*, *Th*. *intermedium*, and *Th*. *ponticum* was labeled with fluorescein-12-dUTP, while St genomic DNA from diploid and tetraploid *P. spicata* was labeled with Texas Red-5-Dutp, and used as GISH and mc-GISH probes. The sheared DNA of CS was used as a blocking DNA. The oligonucleotide probes combination of Oligo-pTa535 (red) and Oligo-pSc119.2 (green) were used for FISH analyses. Hybridization signals were observed and acquired with an Olympus BX53 fluorescence microscope.

### Wheat 15 K SNP array analysis

Wheat 15 K SNP genotyping arrays were used to genotype nine samples, including Abbondanza, ES-9, ES-10, ES-23, ES-24, ES-25, ES-26, *Th. ponticum*, and *Th. intermedium*, using Illumina SNP genotyping technology (China Golden Marker Biotechnology Company). There were 13,199 SNP loci contained in the wheat 15 K array and distributed on all 21 wheat chromosomes. Percentages of the same genotypes in each chromosome between two materials were obtained by calculating the rate of the same genotype loci number in total number of markers. The software Origin (OriginLab, USA) was used for data analysis and graphing.

### PLUG markers analysis

The polymerase chain reaction (PCR)–based landmark unique gene (PLUG) markers (http://wheat.pw.usda.gov/SNP/new/pcr_primers.shtml) were selected for 21 wheat chromosomes among homoeologous groups 1 to 7 and then synthesized by AuGCT DNA-SYN Biotechnology Co. (Beijing, China). PCR assays and electrophoresis procedures were conducted as described before [65].

### Agronomic traits and stripe rust resistance evaluation

The stripe rust resistance evaluation was conducted annually in the field at the adult stage, while the seedling stage test was conducted in the greenhouse in 2020 and 2021. In 2020, a mixture of *Pst* races CYR31 and CYR32 was used to evaluate the adult plant resistance of Abbondanza, ES-9, ES-10, ES-23, ES-24, ES-25, ES-26, Xiaoyan784, and Zhong4, with HXH as susceptible control. Ten plants of each material were evaluated and scored. For further genetic analyses of the resistance, *Pst* race CYR32 was used to inoculate the above-mentioned materials at the seedling stage in 2020 and 2021 with two replicates (five plants of each material were planted and evaluated as one replicate), while the BC_1_F_2_ population individuals of ES-24 and HXH were tested in 2021 with no replication. The infection type (IT) was scored with a scale of 0–4 [66].

To assess the morphological traits, ten plants of each material (Abbondanza, ES-9, ES-10, ES-23, ES-24, ES-25, ES-26, Xiaoyan784, and Zhong4) at the physiological maturity stage were randomly selected during the 2019–2020 growing season. Seven agronomic traits recorded in the field, including plant height, spike length (main spike), number of spikelets per main spike, number of tillers, number of seeds per main spikelets, awnedness, and thousand-kernel weight. The significant differences of each agronomic trait were analyzed by Duncan’s multiple range test (*P* < 0.05).

### Meiotic chromosome pairing analysis of the F_1_ hybrids

Young spikes of F_1_ hybrids derived from the two crosses (ES-9 × ES-23 and ES-10 × ES-24) at appropriate stages were extracted at the suitable temperature under field conditions, and immediately treated with Carnoy’s fixative fluid II (6:3:1 ethanol-chloroform-glacial acetic acid solution). Before cytological observation of pollen mother cells, anthers were extracted and stained with 1% acetocarmine. The chromosome configurations in the meiosis period were observed, recorded and photographed.

### Genomic polymorphism analysis by pairwise comparisons

On the basis of SLAF-seq [67], genomic DNA of Abbondanza, ES-9, ES-10, ES-23, ES-24, *Th. intermedium*, and *Th. ponticum* was sequencedby Biomarker Technologies Co. (Beijing, China). The restriction endonuclease *Hae*III was selected to digest the genomic DNA. According to the sequence similarity, the filtered SLAF pair-end reads (150 bp per read) were clustered. Using BLAST software, sequences with over 90% identity were divided into one SLAF locus. Genomic polymorphism analyses were conducted in two groups, ES-9 and ES-23 (group 1), as well as ES-10 and ES-24 (group 2). First, all the SLAFs from the two groups were blasted with the wheat genome, and the sequences with high wheat homology (over 80%) were removed. Second, the remaining SLAFs were further blasted with the sequences of *Th. ponticum* or *Th. intermedium*. The SLAFs with high identity (over 90%) remained, and served as specific sequences of *Th. ponticum* attributed to ES-9 and ES-10, as well as the specific sequences of *Th. intermedium* attributed to ES-23 and ES-24. Finally, intercomparisons within groups were conducted and the specific SLAFs with high identity (over 90%) were acquired.

### Development and validation of the St-chromosome-specific markers

Based on the obtained specific SLAFs, PCR primers were designed to amplify the two groups of materials. All the primers were designed using the online tool Primer3 Plus (http://www.bioinformatics.nl/cgi-bin/primer3plus/primer3plus.cgi) and synthesized by AuGCT DNA-SYN Biotechnology Co. (Beijing, China). The amplified products were examined using 2% agarose gel electrophoresis. The markers amplified specific sequences in tetraploid *P. spicata*, diploid *P. spicata*, *Th. ponticum*, *Th. intermedium*, DA2St, ES-9, and ES-23, but not in CS, Abbondanza, *Th. bessarabicum*, *Th. elongatum*, the 1St and 3-7St addition lines, and served as 2St-chromosome-specific molecular markers. The markers present in ES-10 and ES-24, but absent in the 1-2St and 4-7St addition lines, served as 3St-chromosome-specific molecular markers. Subsequently, the 3St-chromosomes-specific markers were utilized in BC_1_F_2_ individuals of ES-24 and HXH for further genetic analysis.

PCR amplifications were performed in a reaction of 20 μl, containing 1.6 μl of template DNA (100 ng/µl), 1.6 μl dNTP mixture (2.5 mM each), 2 μl of 10 × PCR buffer (Mg^2+^ plus), 1.4 μl of each primer (10 µM), 0.1 μl *rTaq* DNA polymerase (2.5 U/μl, Takara), and 13.3 μl double-distilled water. The PCR protocol was as follows: 94 °C for 4 min; 32 cycles of 94 °C for 30 s, 54–60 °C for 35 s, 72 °C for 30 s, and 72 °C for 30 s; 72 °C for 10 min.

## Supplementary Information

**Additional file 1: Fig. S1.** Uncropped images of Fig. 1.

**Additional file 2: Table S1.** SNP array raw data of the six substitution lines for Fig. 2.

**Additional file 3: Table S2.**  PLUG polymorphic markers mapped on homoeologous group 2 and 3 used to linkage analysis of Thinopyrum ponticum and Thinopyrum intermedium chromosome.

**Additional file 4: Fig. S2.** Uncropped gel images of markers of Fig. 3.

**Additional file 5: Fig. S3.** Raw images of  Fig. 4 and ES-12.

**Additional file 6: Fig. S4.** The crossing program of wheat-Thinopyrum substitution lines.

**Additional file 7: Table S3.** Quality of SLAF data.

**Additional file 8: Fig. S5.** Uncropped gel images of markers of Fig. 6.

**Additional file 9: Fig. S6.** Uncropped gel images of markers of Fig. 8.

## Data Availability

The datasets used and/or materials and/or codes during the current study are available from the corresponding author on reasonable request. The data sets were deposited in the China National Center for Bioinformation (CNCB) database under accession number PRJCA006783.
