# Supplementary material for: Molecular cytogenetics and development of St-chromosome-specific molecular markers of novel stripe rust resistant wheat–Thinopyrum intermedium and wheat–Thinopyrum ponticum substitution lines
Source: BMC Plant Biol. 2022 Mar 12;22:111. doi: 10.1186/s12870-022-03496-x (PMC8917741; doi:10.1186/s12870-022-03496-x)
Supplement: Supplementary file 1 — Additional file 1: Fig. S1. Uncropped images of Fig. 1. [file 12870_2022_3496_MOESM1_ESM.pdf]

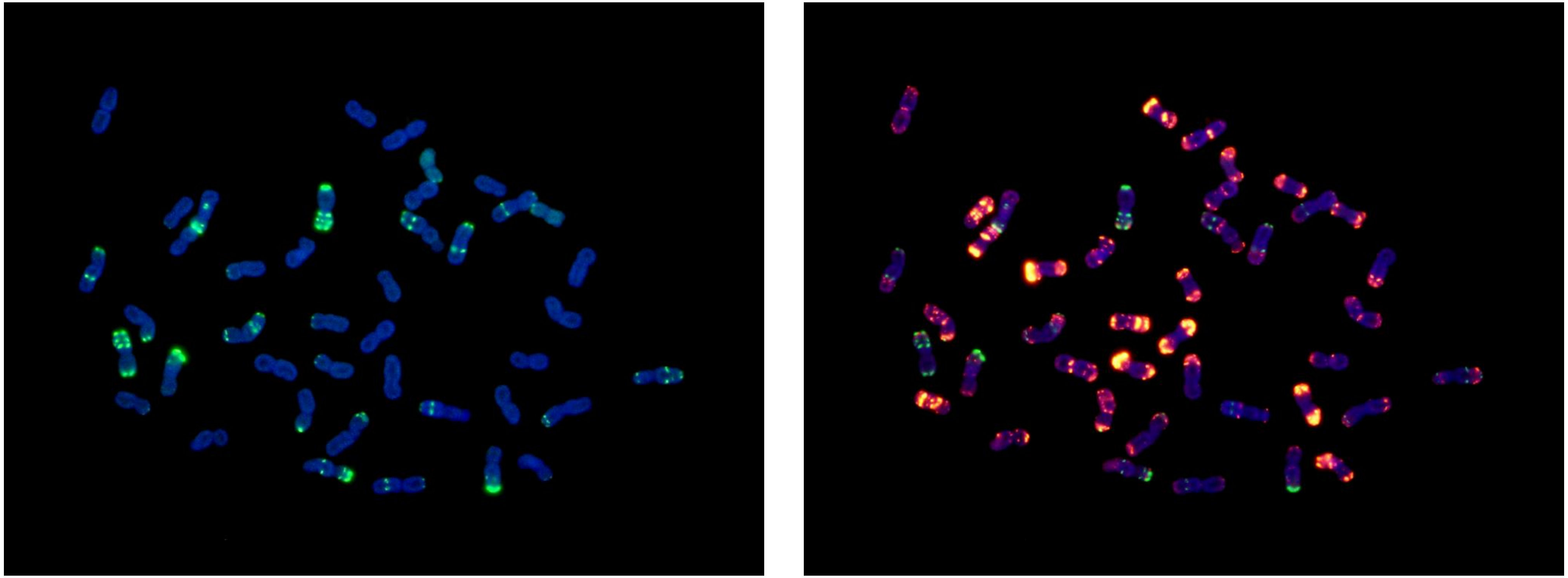

Uncropped images of a1 and b1 in Fig. 1

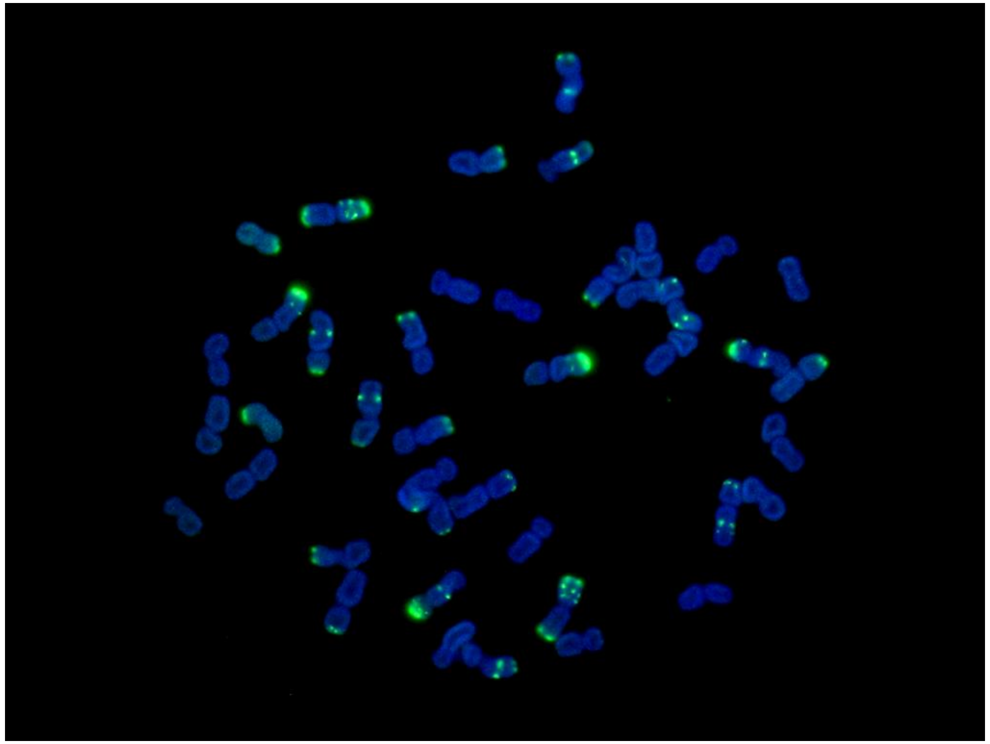

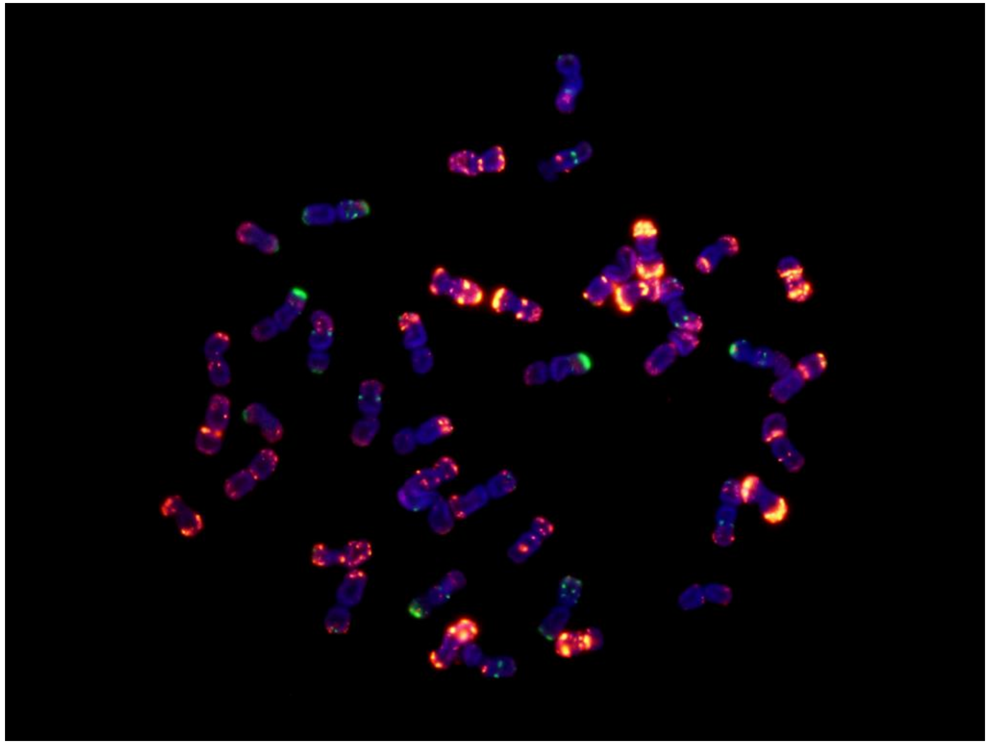

Uncropped images of a2 and b in Fig. 1

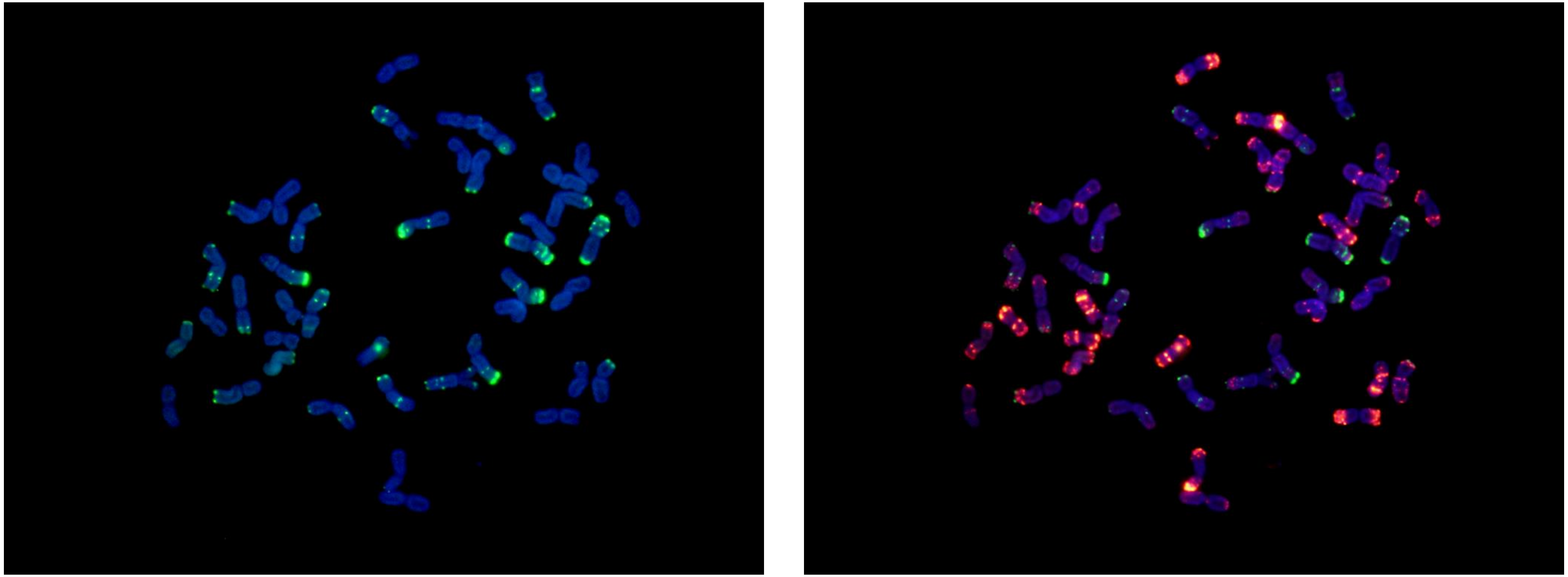

Uncropped images of a3 and b3 in Fig. 1

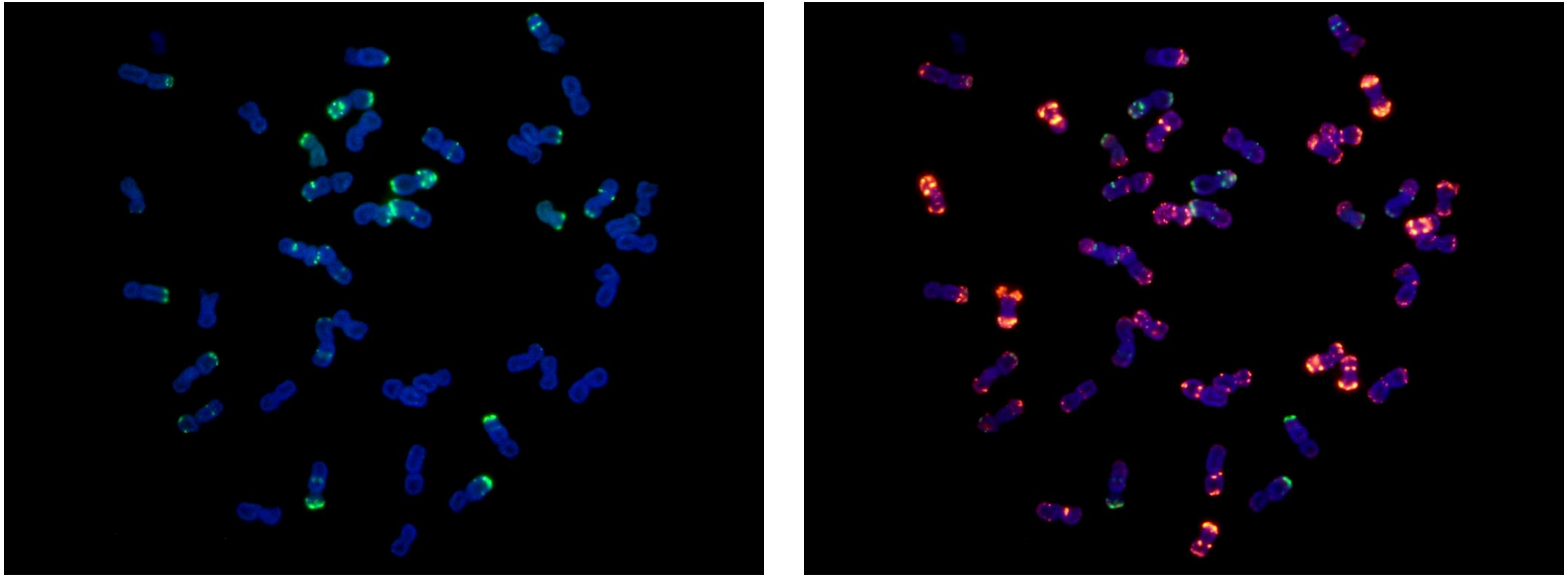

Uncropped images of a4 and b4 in Fig. 1

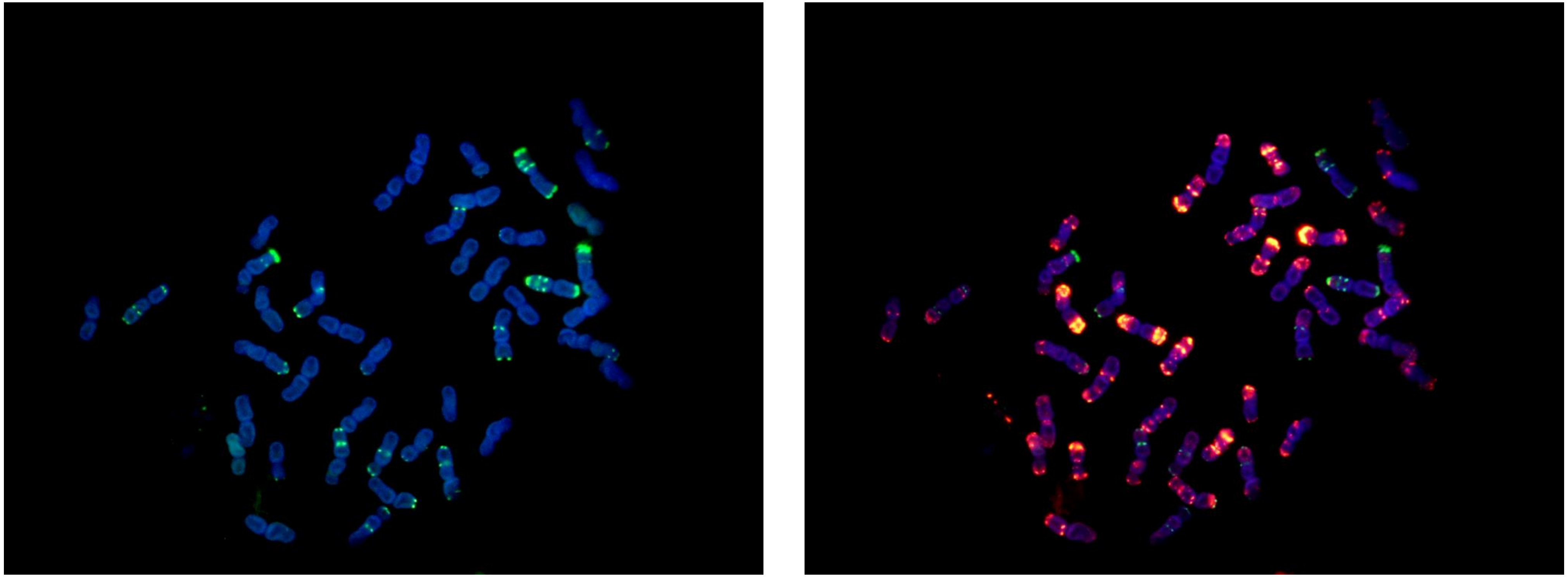

Uncropped images of a5 and b5 in Fig. 1

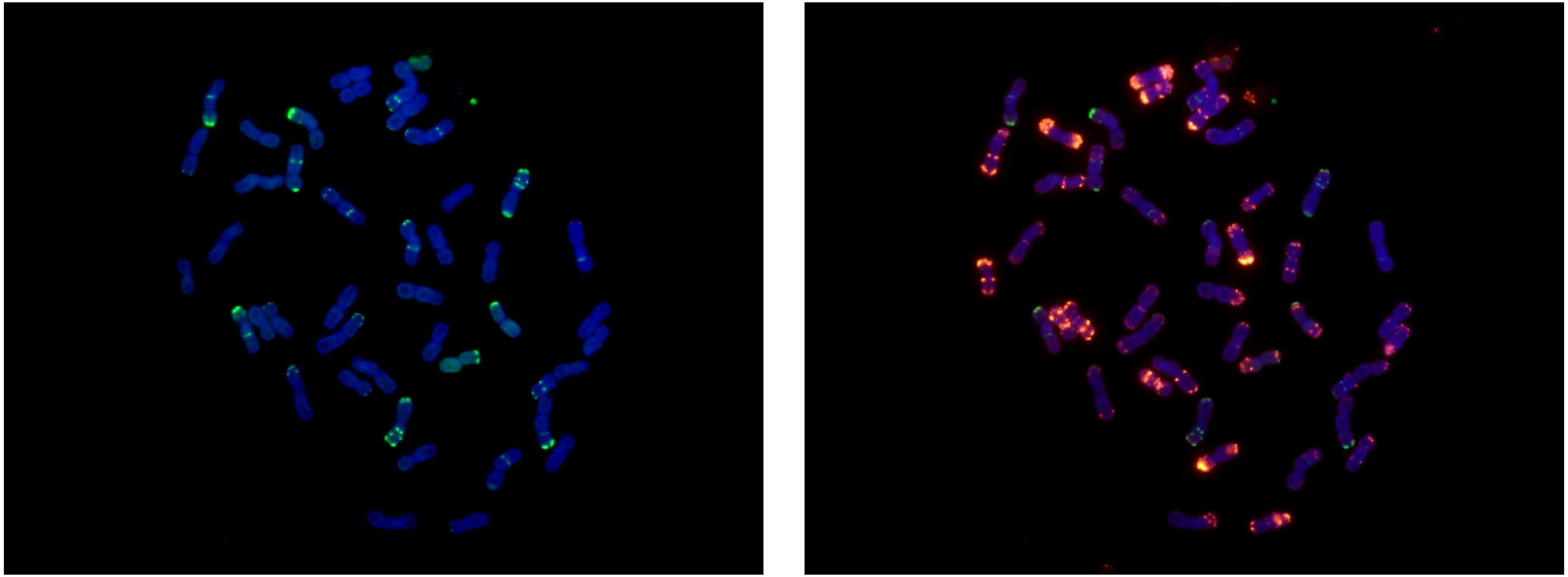

Uncropped images of a6 and b6 in Fig. 1

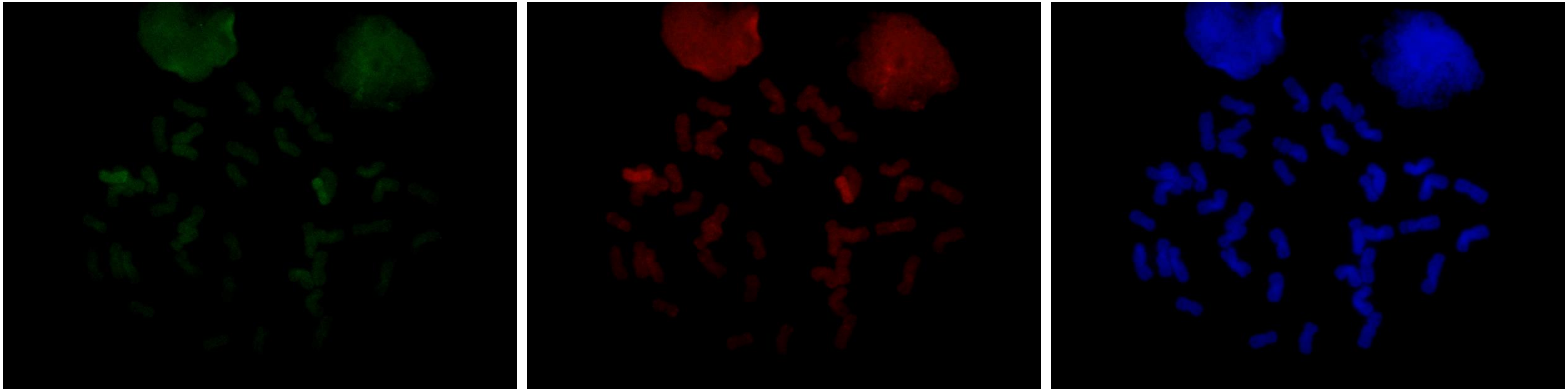

Raw images of c1 in Fig. 1

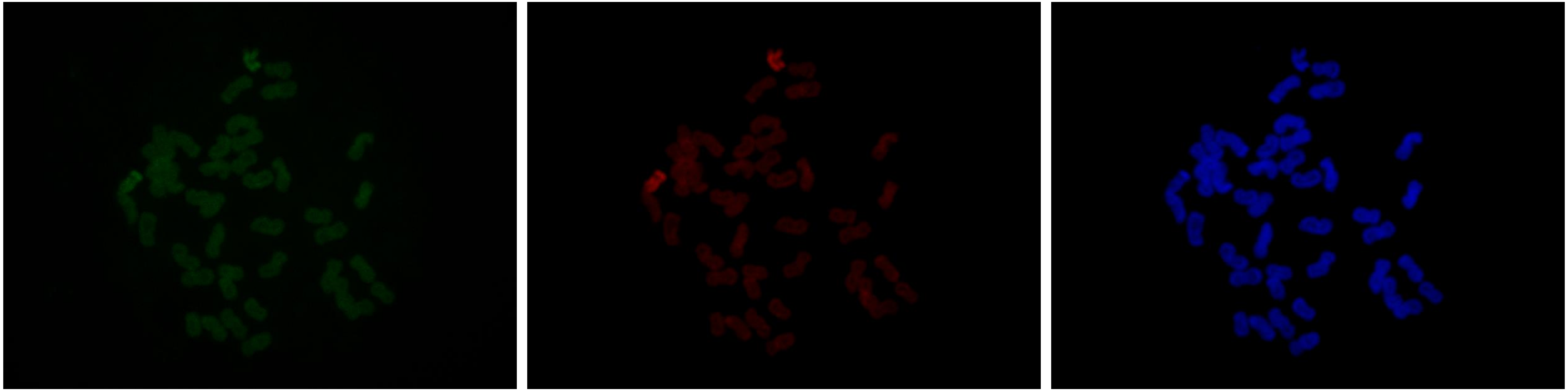

Raw images of c2 in Fig. 1

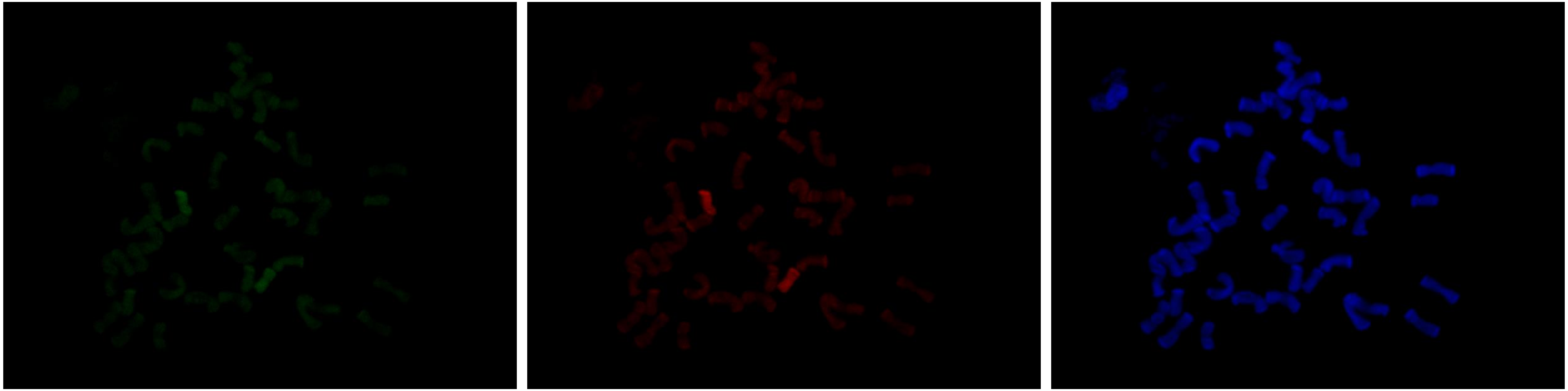

Raw images of c3 in Fig. 1

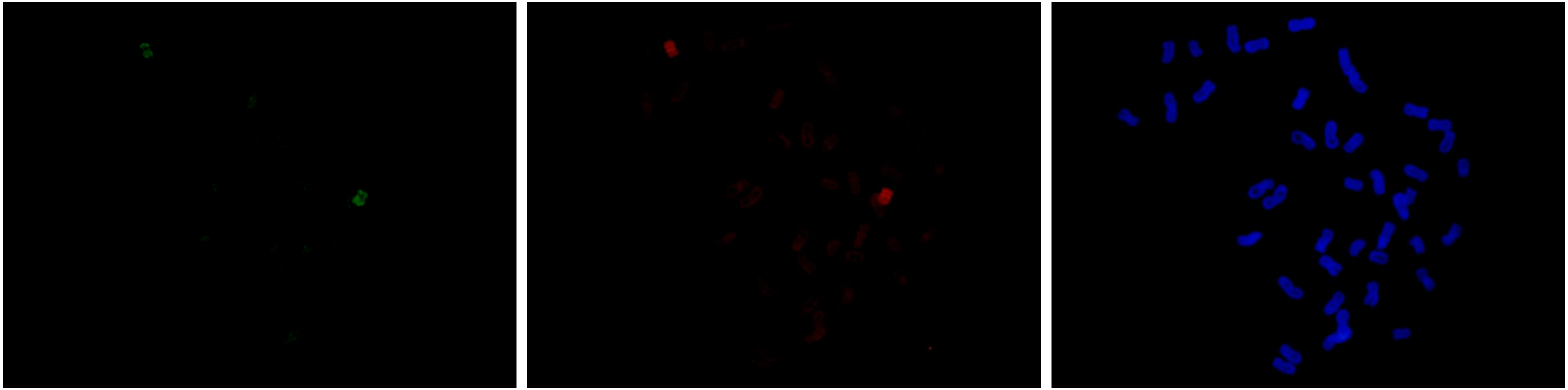

Raw images of c4 in Fig. 1

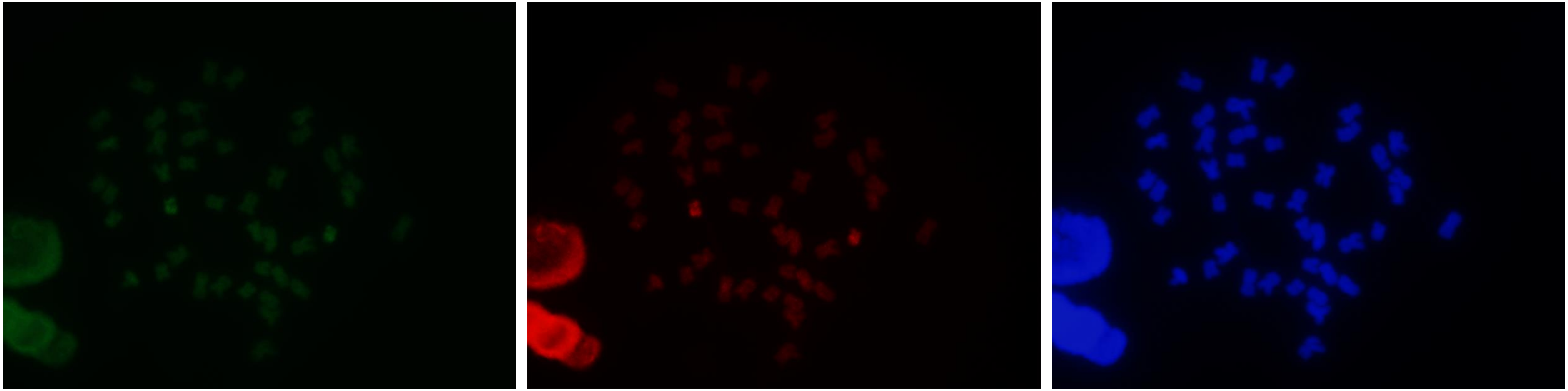

Raw images of c5 in Fig. 1

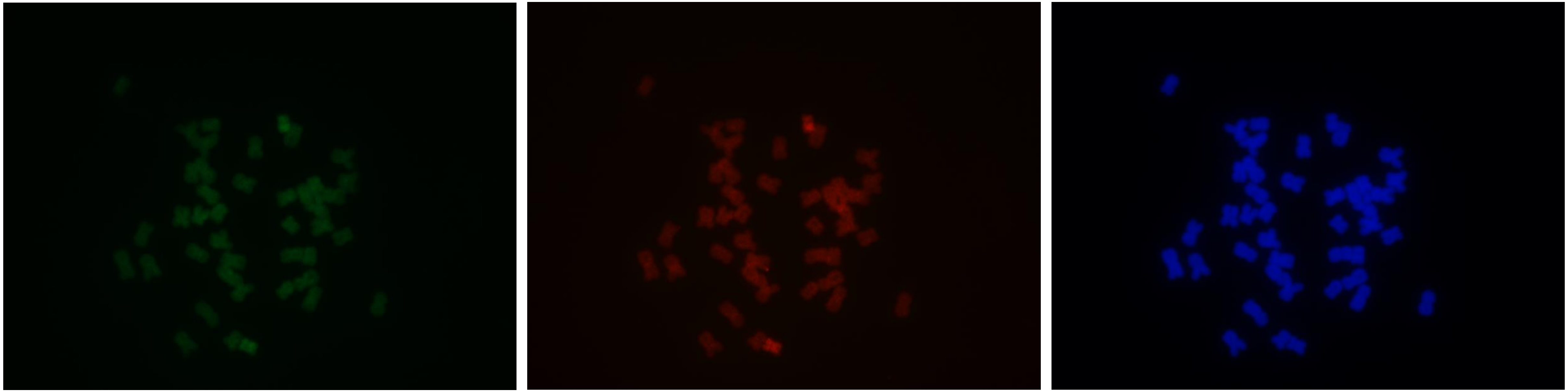

Raw images of c6 in Fig. 1
