# Supplementary material for: Molecular cytogenetics and development of St-chromosome-specific molecular markers of novel stripe rust resistant wheat–Thinopyrum intermedium and wheat–Thinopyrum ponticum substitution lines
Source: BMC Plant Biol. 2022 Mar 12;22:111. doi: 10.1186/s12870-022-03496-x (PMC8917741; doi:10.1186/s12870-022-03496-x)
Supplement: Supplementary file 3 — Additional file 3: Table S2. PLUG polymorphic markers mapped on homoeologous group 2 and 3 used to linkage analysis of Thinopyrum ponticum and Thinopyrum intermedium chromosome. [file 12870_2022_3496_MOESM3_ESM.pdf]

Table S2. PLUG polymorphic markers mapped on homoeologous group 2 and 3 used to linkage analysis of *Thinopyrum ponticum* and *Thinopyrum intermedium* chromosome.

Markers	Type	Primers (5'-3')	Location	Gel type/Restriction enzyme	Tm (℃)/ time of enzyme digesttion (h)
TNAC1142	PLUG	F: GCCTACGAGTACATGGTCGAG<br>R: CAGCATCCATAACCAGGATGT	2AL 2BL 2DL	1.5% agarose gel/ *Taq* Ⅰ /*Hae*Ⅲ	60/ 2 or 3
TNAC1132	PLUG	F: TATTGGTAGCCTTGTCGCTCT<br>R: TATGCTGCATGTGCTATCGAC	2AL 2BL 2DL	1.5% agarose gel/*Taq* Ⅰ	60/ 2
TNAC1140	PLUG	F: TCCCAGAAATTACAAGGCTCA<br>R: AGGAACCCTATGCATTGGAAA	2AL 2BL 2DL	1.5% agarose gel/*Taq* Ⅰ	60/ 2
TNAC1326	PLUG	F: ACAGATCGAGATGTTTATTGAAA<br>R: GATCAAAGAGATGCGCTGAAG	3AS 3BS 3DS	1.5% agarose gel/*Taq* Ⅰ /*Hae* Ⅲ	60/ 2 or 3
TNAC1359	PLUG	F: GTAAATAGCGCCATCTGCGTA<br>R: CTCTGGATGCAGTTGGAATGT	3AL 3BL 3DL	1.5% agarose gel/*Taq* Ⅰ	60/ 2
