# Supplementary material for: Molecular cytogenetics and development of St-chromosome-specific molecular markers of novel stripe rust resistant wheat–Thinopyrum intermedium and wheat–Thinopyrum ponticum substitution lines
Source: BMC Plant Biol. 2022 Mar 12;22:111. doi: 10.1186/s12870-022-03496-x (PMC8917741; doi:10.1186/s12870-022-03496-x)
Supplement: Supplementary file 4 — Additional file 4: Fig. S2. Uncropped gel images of markers of Fig. 3. [file 12870_2022_3496_MOESM4_ESM.pdf]

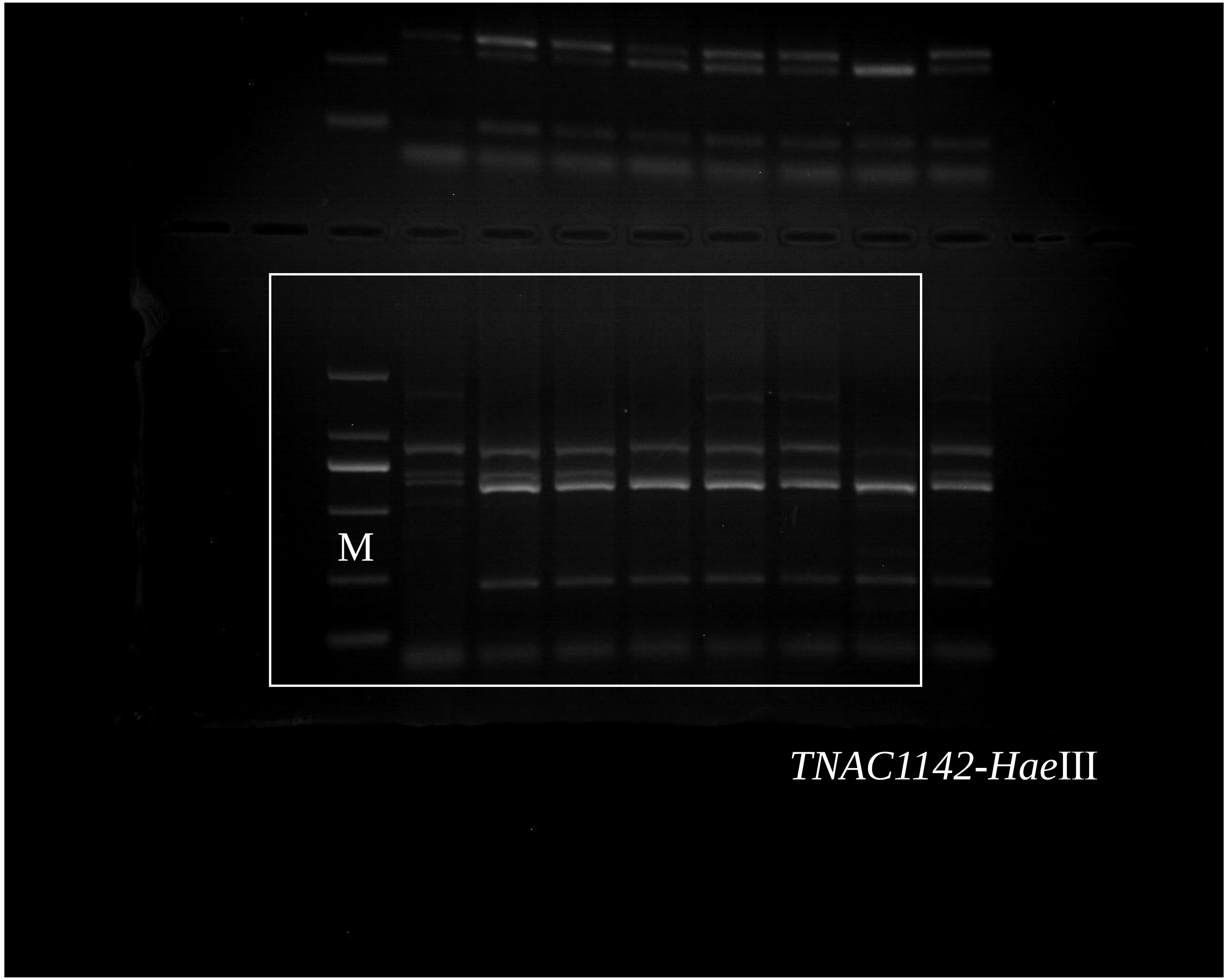

Original gel images of Primer *TNAC1142-Hae*III in Fig. 3

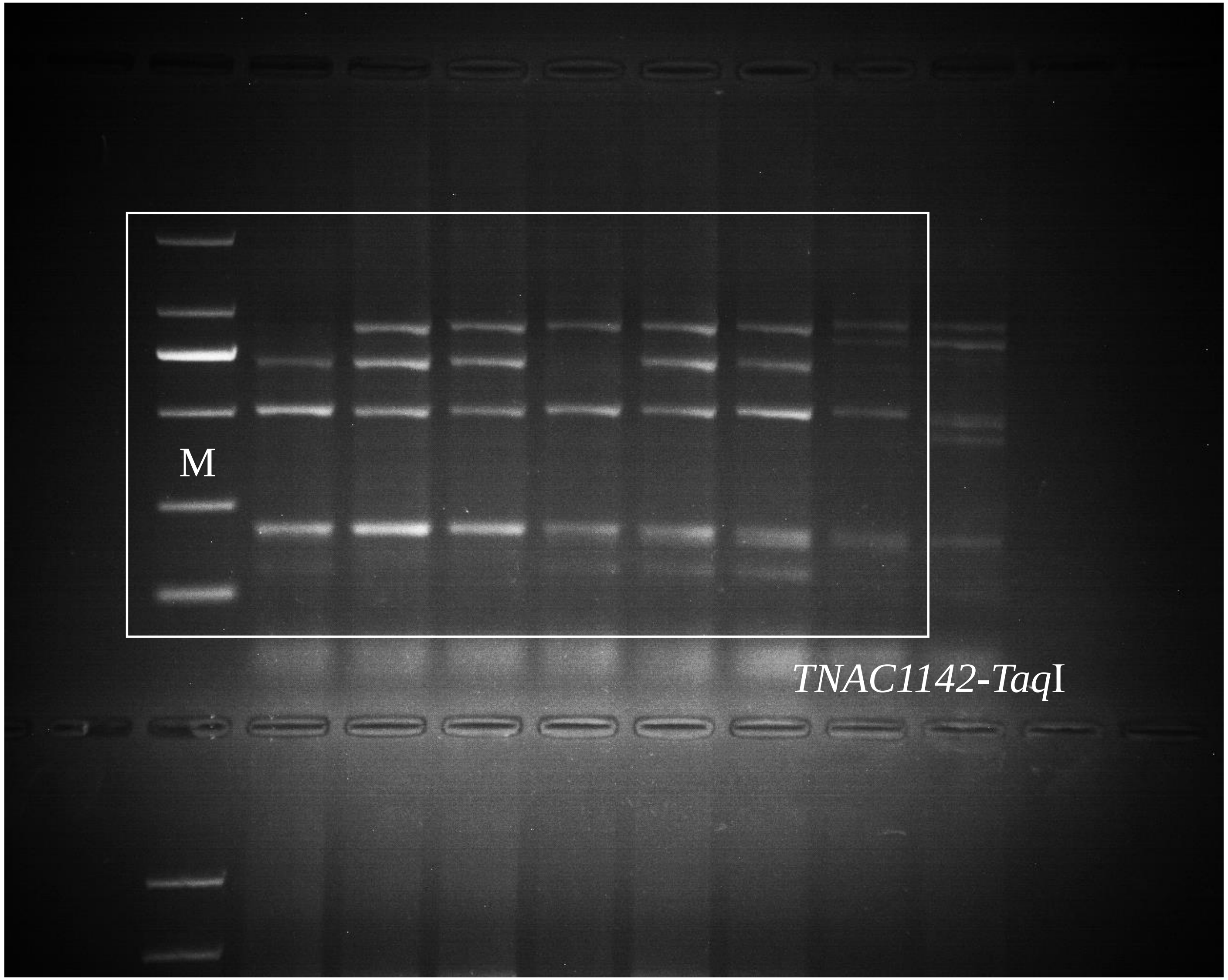

Original gel images of Primer *TNAC1142-Taq*I in Fig. 3

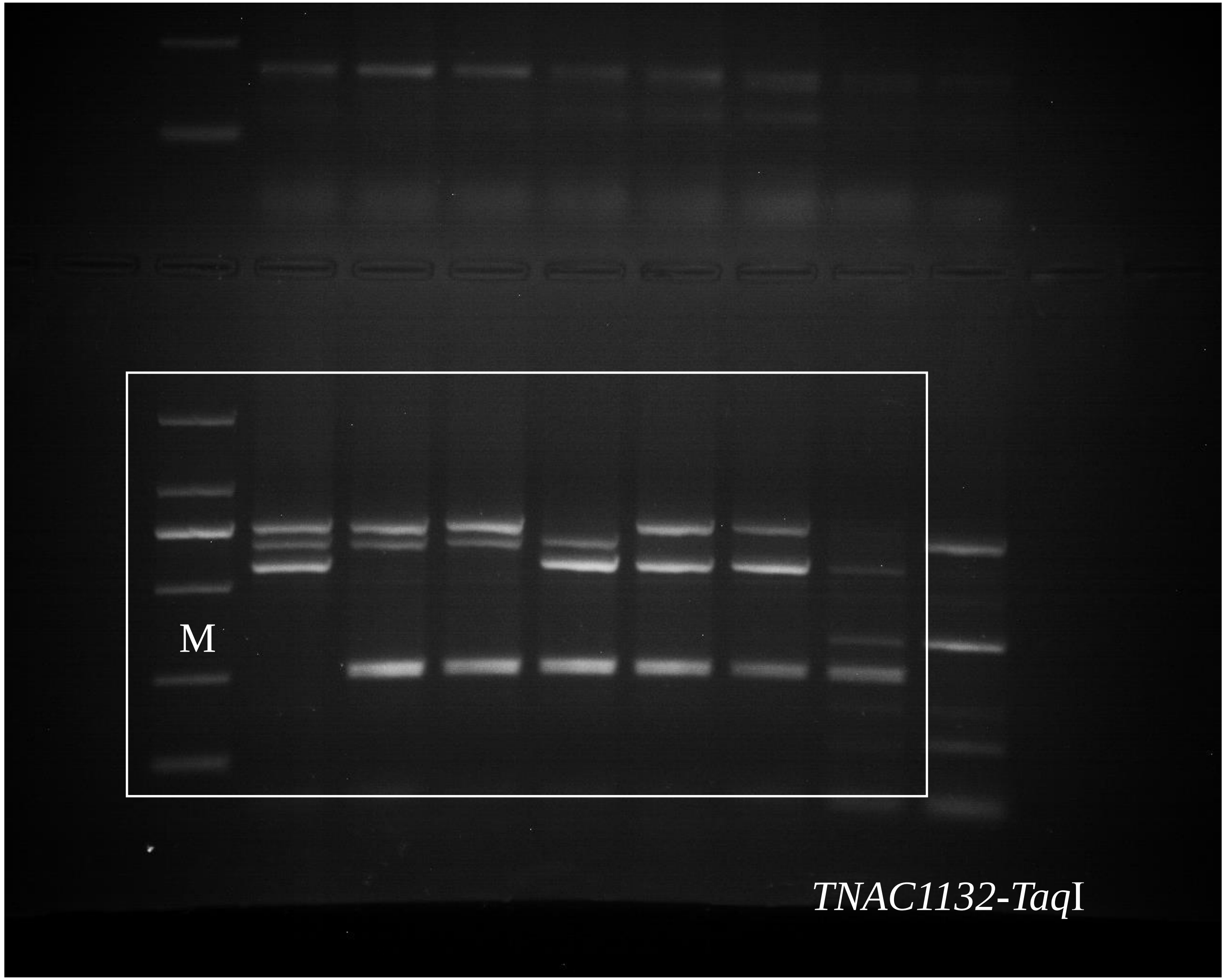

Original gel images of Primer *TNAC1132-Taq*I in Fig. 3

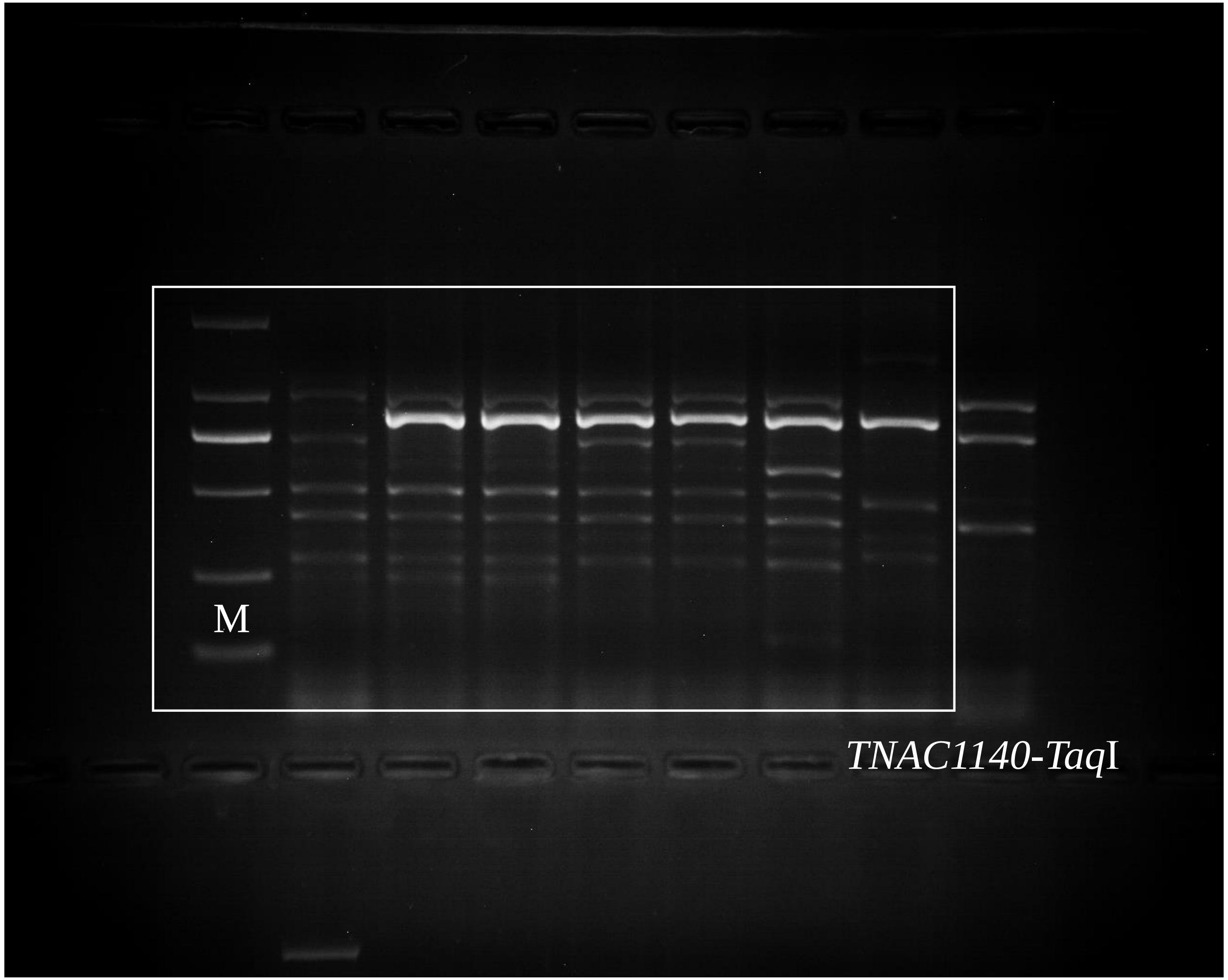

Original gel images of Primer *TNAC1140-Taq*I in Fig. 3

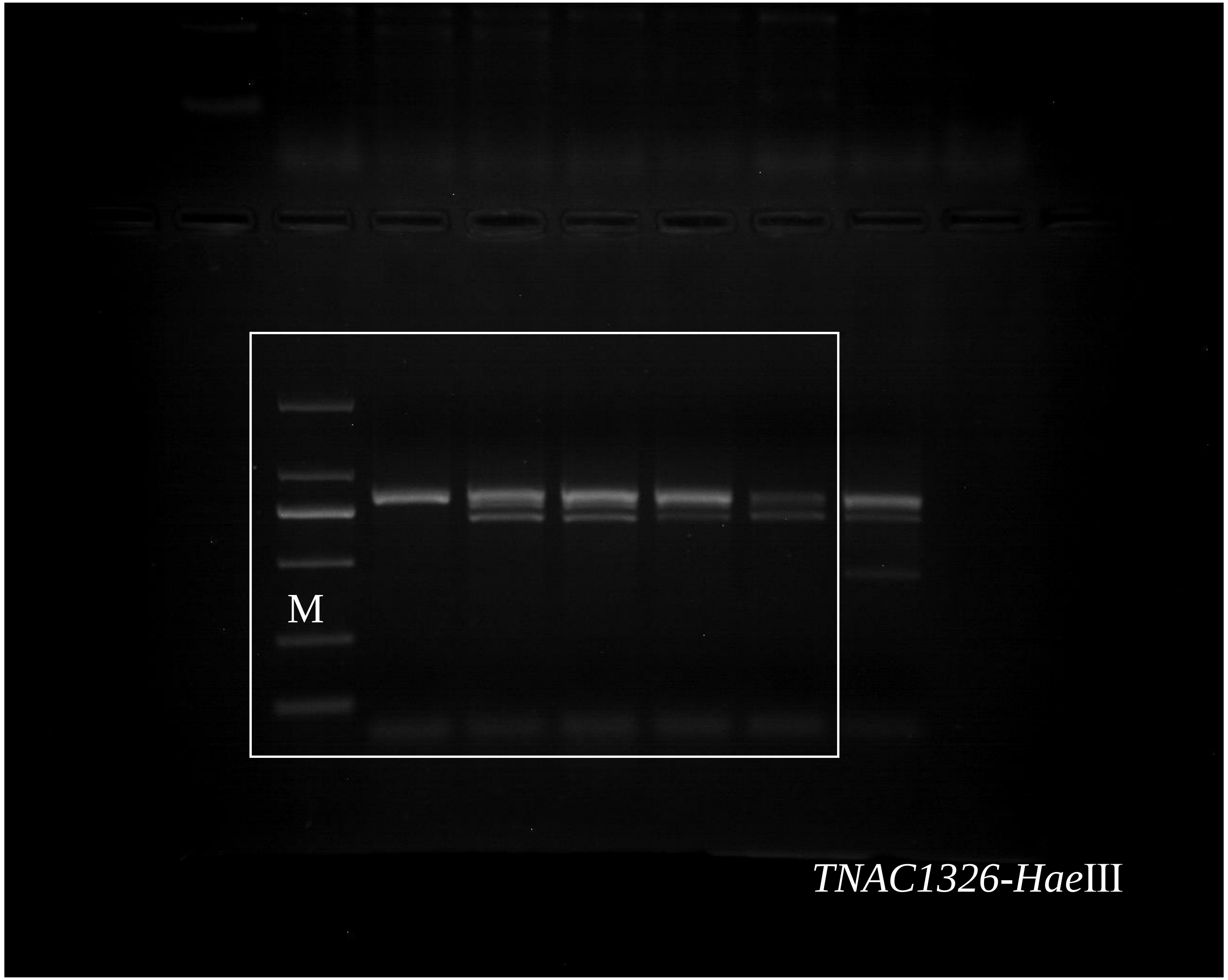

Original gel images of Primer *TNAC1326-Hae*III in Fig. 3

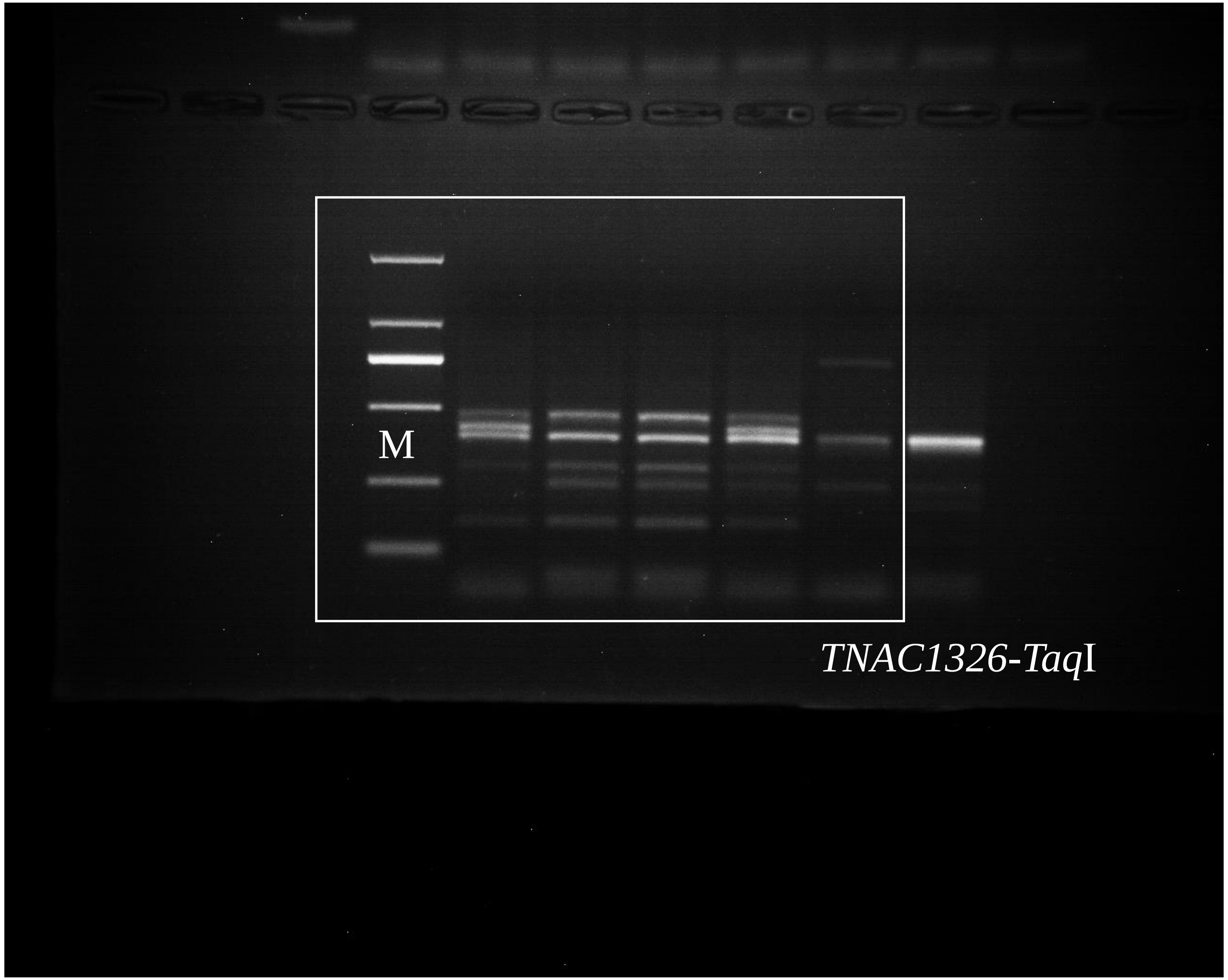

Original gel images of Primer *TNAC1326-Taq*I in Fig. 3

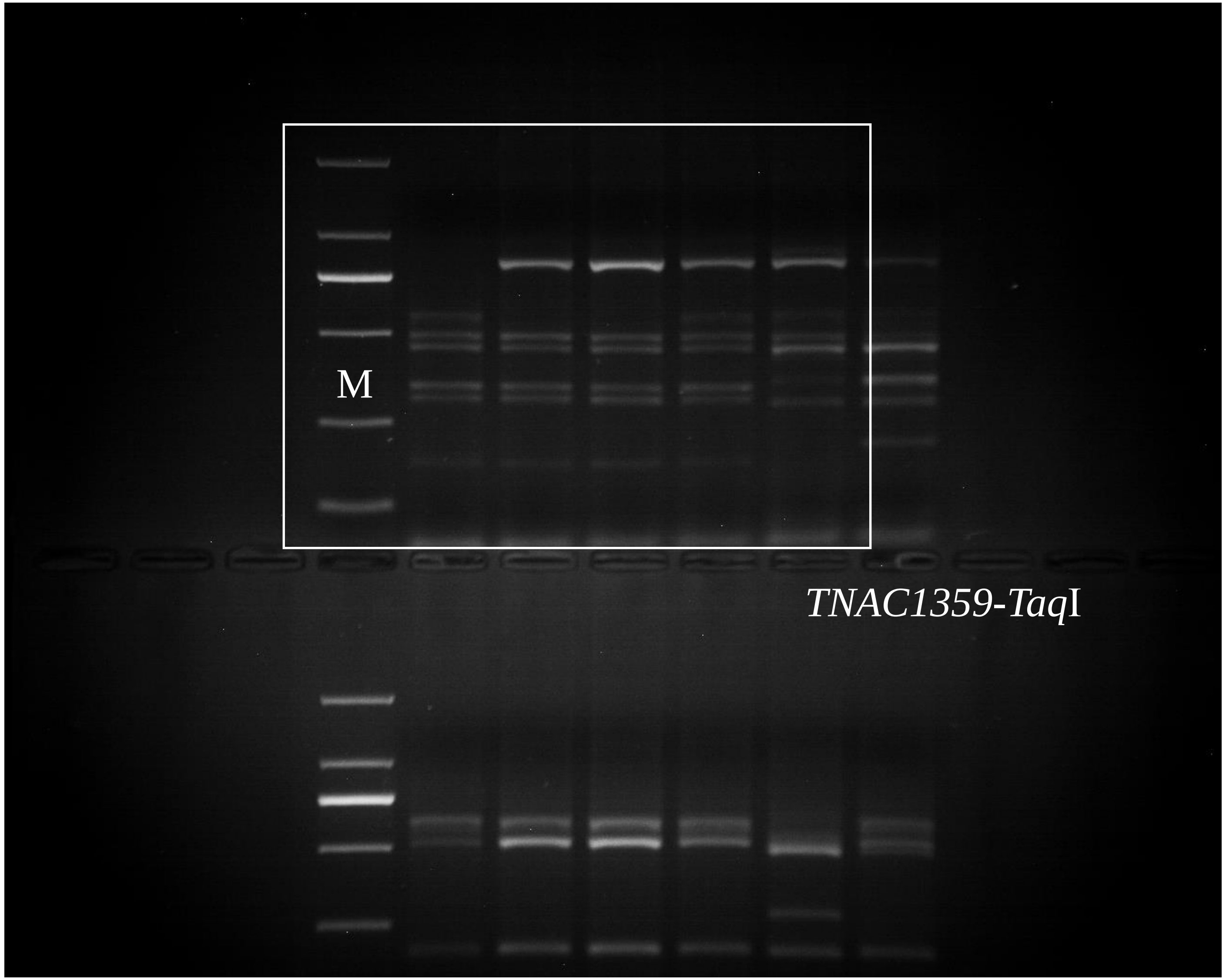

Original gel images of Primer *TNAC1359-Taq*I in Fig. 3
