# Supplementary material for: Molecular cytogenetics and development of St-chromosome-specific molecular markers of novel stripe rust resistant wheat–Thinopyrum intermedium and wheat–Thinopyrum ponticum substitution lines
Source: BMC Plant Biol. 2022 Mar 12;22:111. doi: 10.1186/s12870-022-03496-x (PMC8917741; doi:10.1186/s12870-022-03496-x)
Supplement: Supplementary file 5 — Additional file 5: Fig. S3. Raw images of Fig. 4 and ES-12. [file 12870_2022_3496_MOESM5_ESM.pdf]

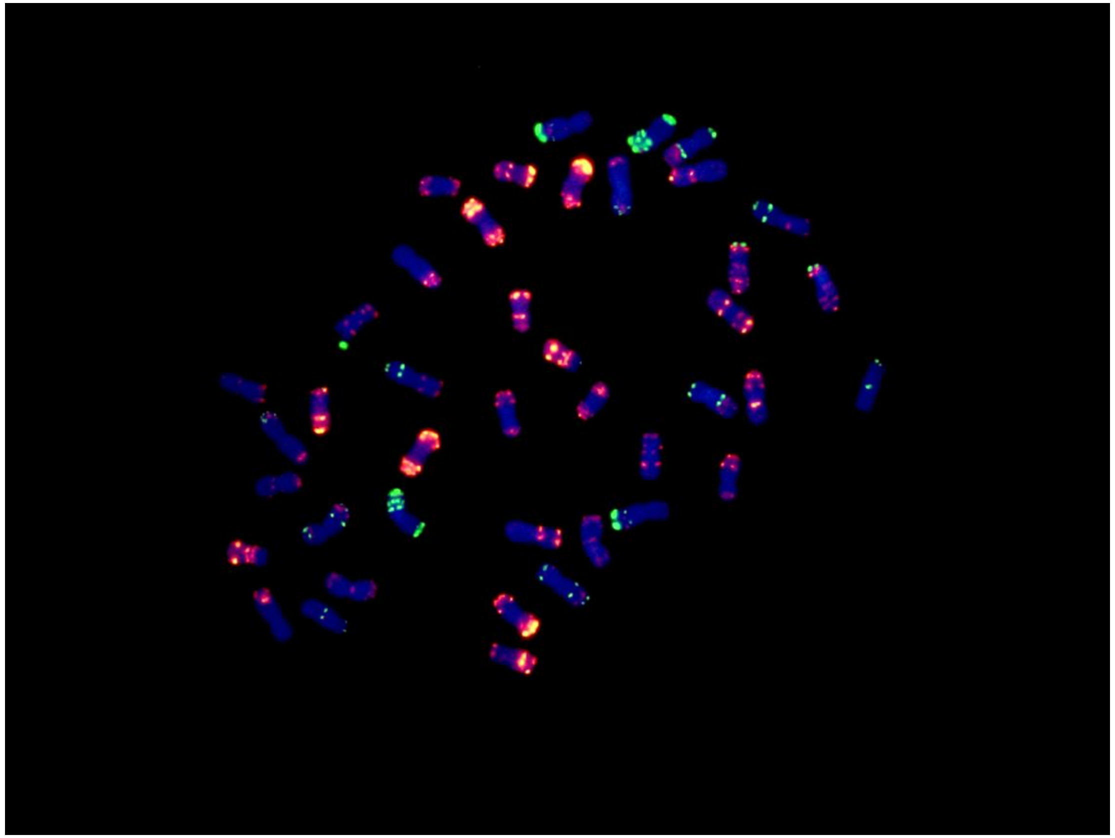

Raw images of a1 in Fig. 4

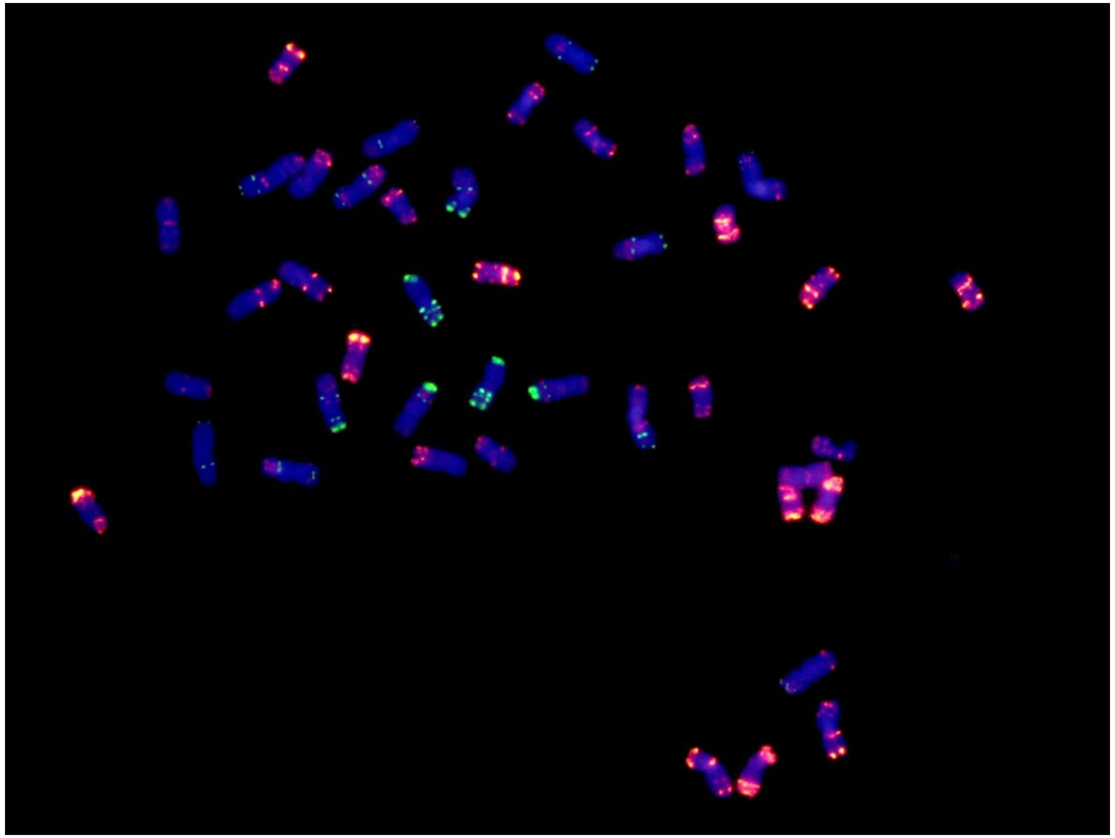

Raw images of b1 in Fig. 4

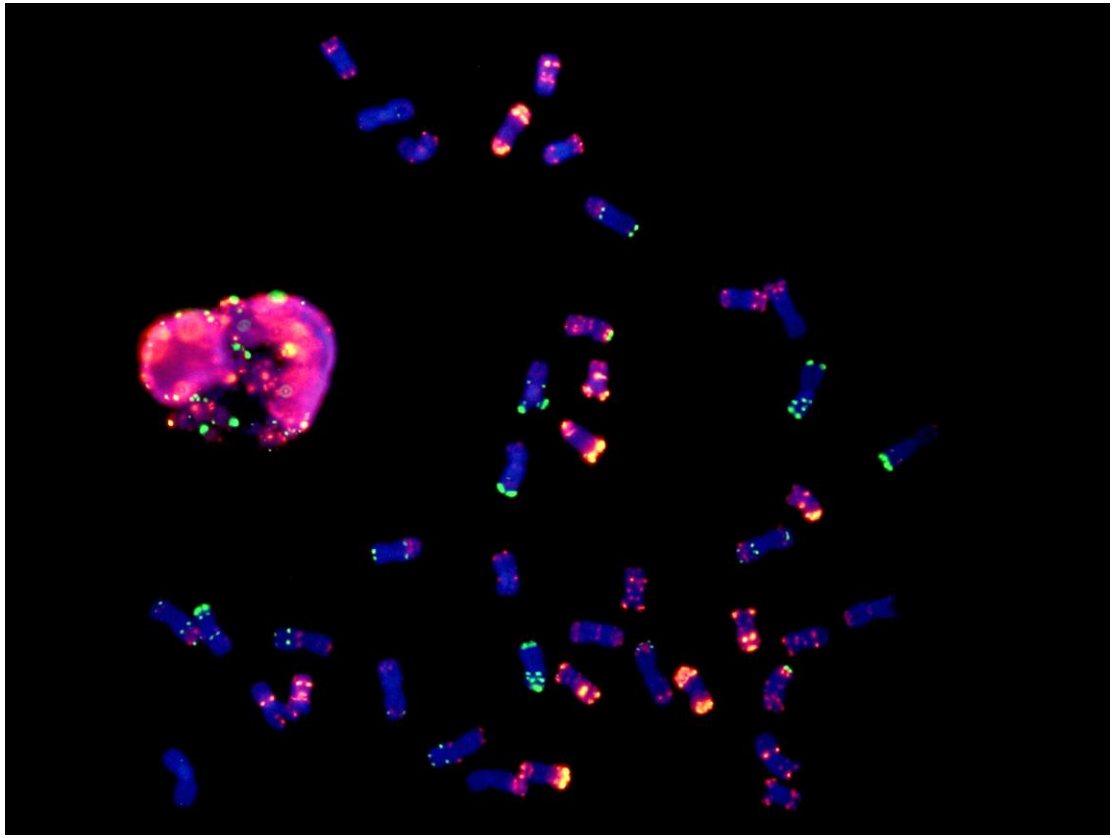

Raw images of c1 in Fig. 4

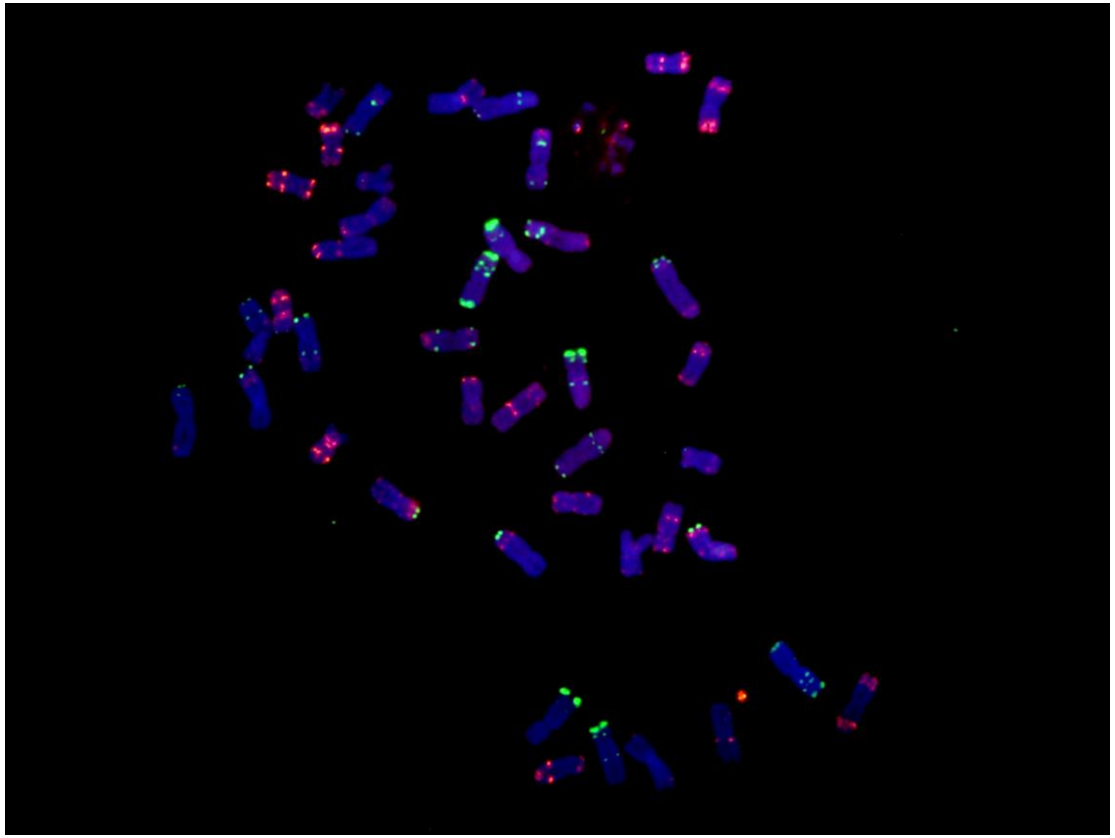

Raw images of d1 in Fig. 4

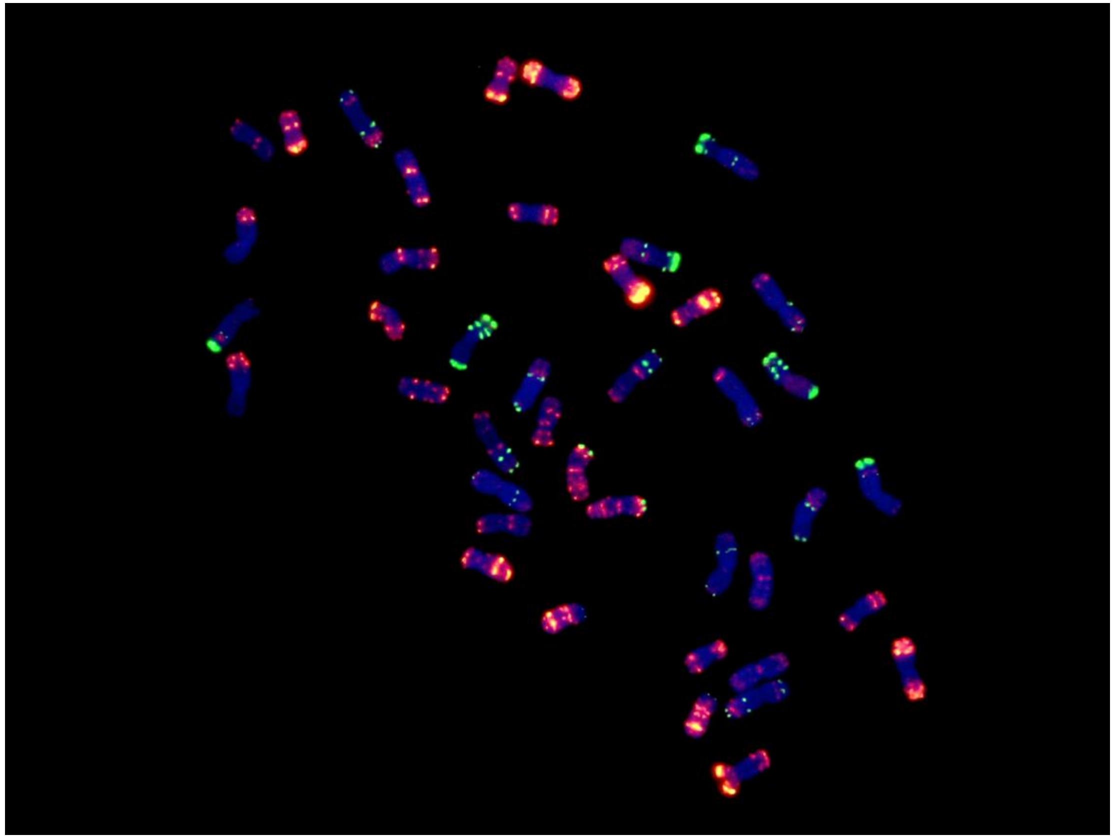

Raw images of e1 in Fig. 4

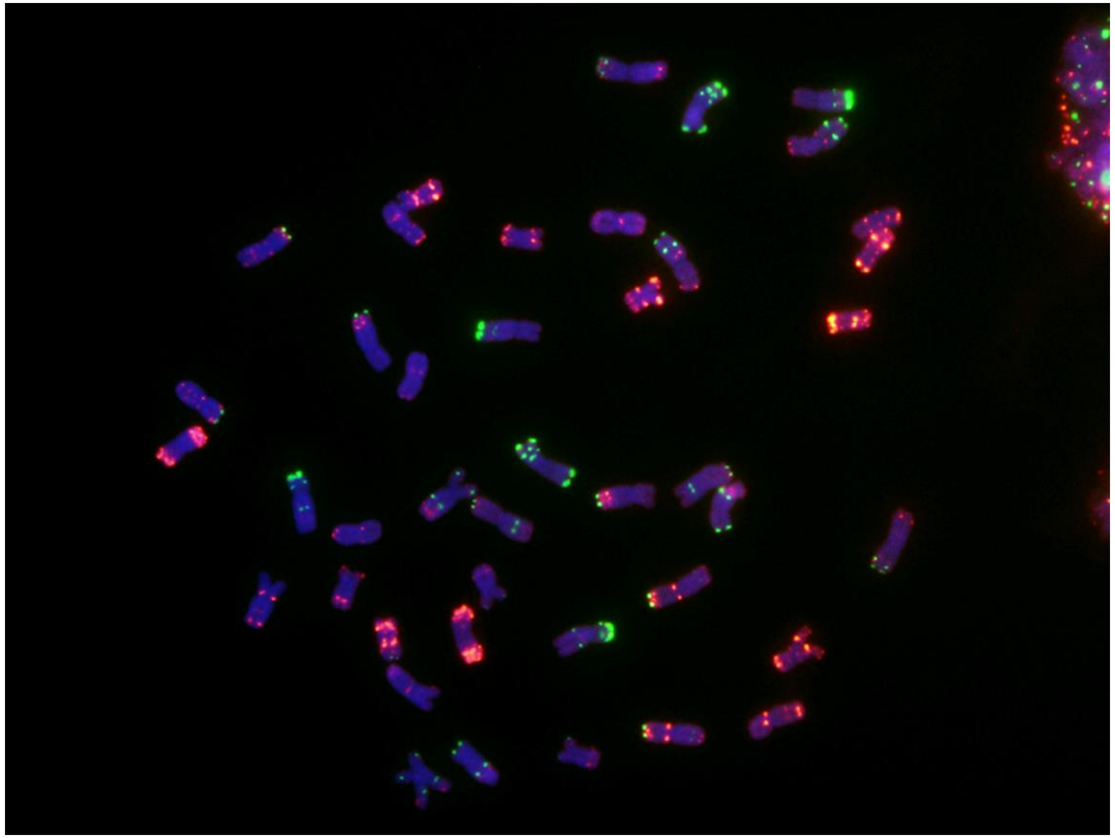

Raw images of f1 (ES-10) in Fig. 4

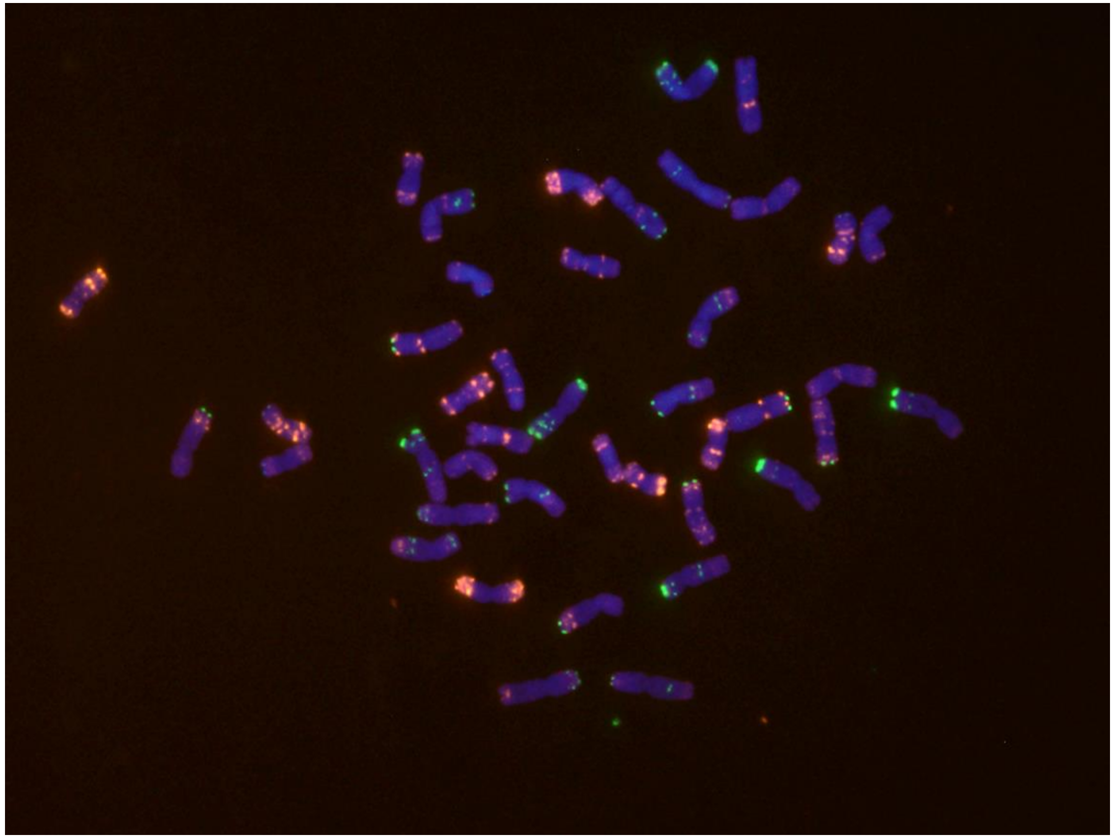

Raw images of ES-12 FISH analyses
