# Supplementary material for: Molecular cytogenetics and development of St-chromosome-specific molecular markers of novel stripe rust resistant wheat–Thinopyrum intermedium and wheat–Thinopyrum ponticum substitution lines
Source: BMC Plant Biol. 2022 Mar 12;22:111. doi: 10.1186/s12870-022-03496-x (PMC8917741; doi:10.1186/s12870-022-03496-x)
Supplement: Supplementary file 6 — Additional file 6: Fig. S4. The crossing program of wheat-Thinopyrum substitution lines. [file 12870_2022_3496_MOESM6_ESM.pdf]

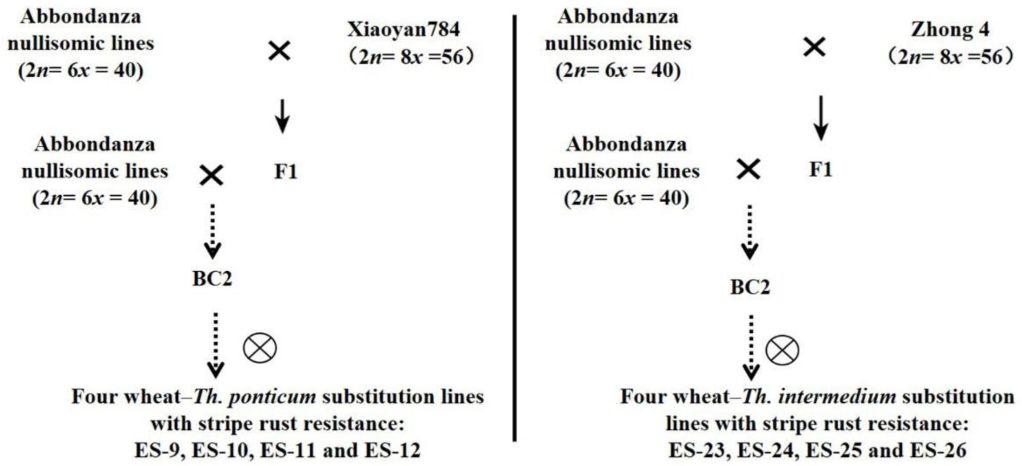

Fig. S4. The crossing program showing strategies employed to create wheat–*Thinopyrum* substitution lines.
