# Supplementary material for: Molecular cytogenetics and development of St-chromosome-specific molecular markers of novel stripe rust resistant wheat–Thinopyrum intermedium and wheat–Thinopyrum ponticum substitution lines
Source: BMC Plant Biol. 2022 Mar 12;22:111. doi: 10.1186/s12870-022-03496-x (PMC8917741; doi:10.1186/s12870-022-03496-x)
Supplement: Supplementary file 7 — Additional file 7: Table S3. Quality of SLAF data. [file 12870_2022_3496_MOESM7_ESM.pdf]

Table S3. Quality of SLAF data.

Genotype	Clean base (bp)	Clean reads	Q20(%)	Q30(%)	GC(%)
**ES-9**	1,277,894,774	6,401,833	97.16	91.82	47.77
**ES-10**	1,054,658,932	5,284,631	96.36	90.29	47.83
**ES-23**	1,906,494,968	9,555,684	97.06	91.55	47.76
**ES-24**	1,228,787,742	6,155,836	97.08	91.59	47.72
**Abbondanza**	1,297,710,674	6,495,947	96.31	90.13	47.65
Thinopyrum ponticum	1,520,267,864	7,613,789	96.84	90.98	47.45
Thinopyrum intermedium	2,038,483,374	10,205,879	97.10	91.50	46.77
