# Supplementary material for: Molecular cytogenetics and development of St-chromosome-specific molecular markers of novel stripe rust resistant wheat–Thinopyrum intermedium and wheat–Thinopyrum ponticum substitution lines
Source: BMC Plant Biol. 2022 Mar 12;22:111. doi: 10.1186/s12870-022-03496-x (PMC8917741; doi:10.1186/s12870-022-03496-x)
Supplement: Supplementary file 8 — Additional file 8: Fig. S5. Uncropped gel images of markers of Fig. 6. [file 12870_2022_3496_MOESM8_ESM.pdf]

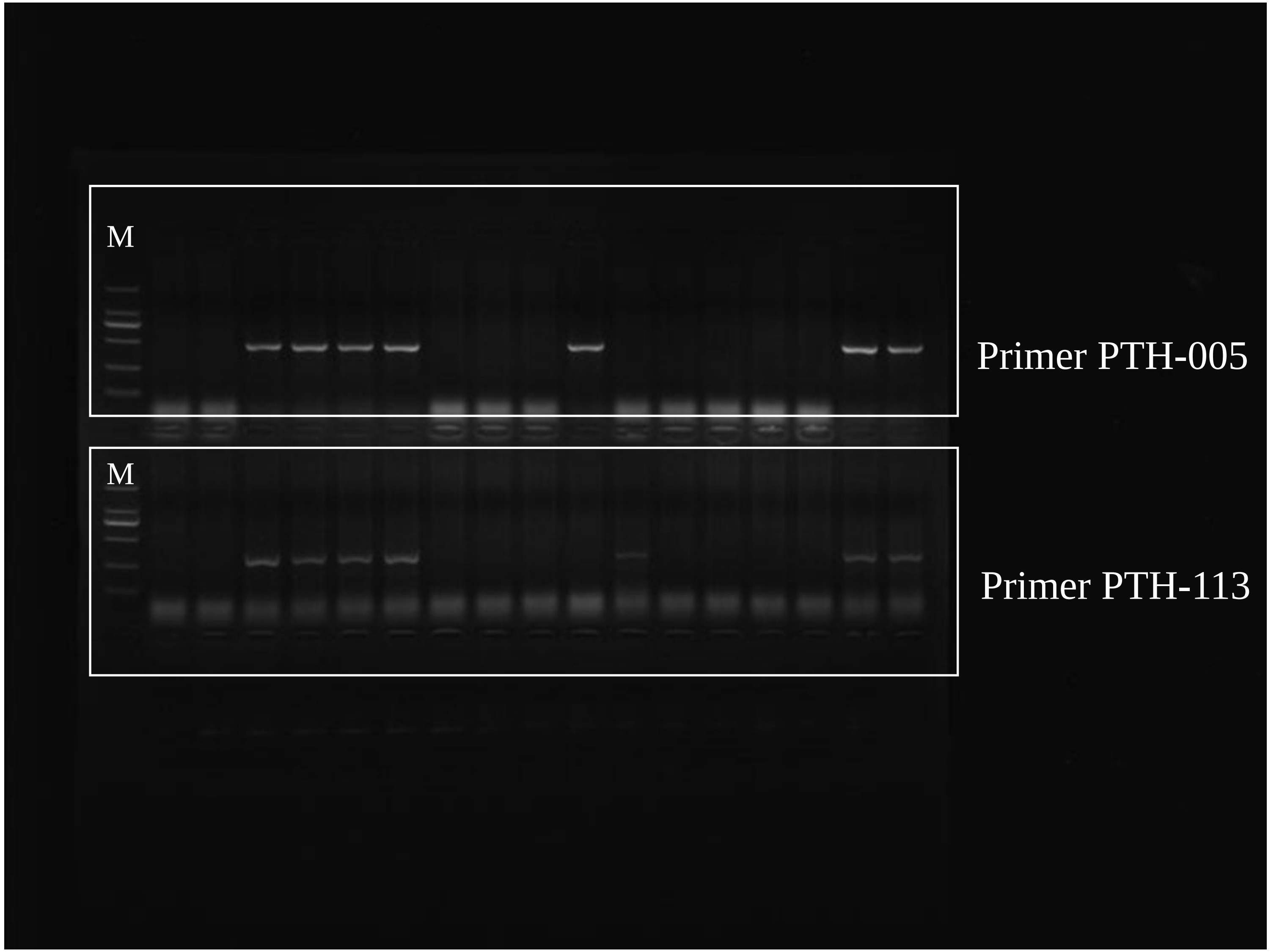

Original gel images of Primer in Fig. 6

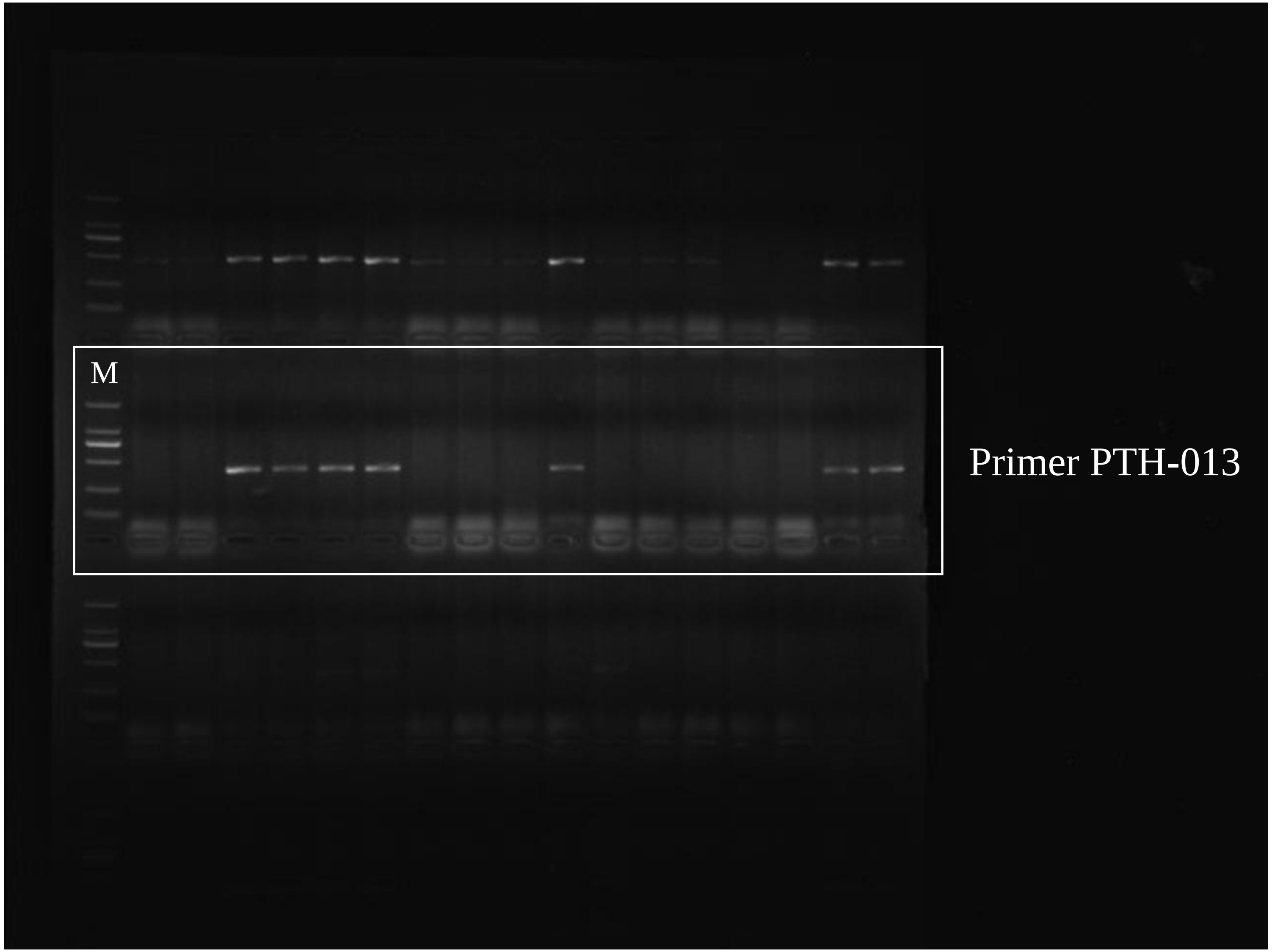

Original gel images of Primer in Fig. 6

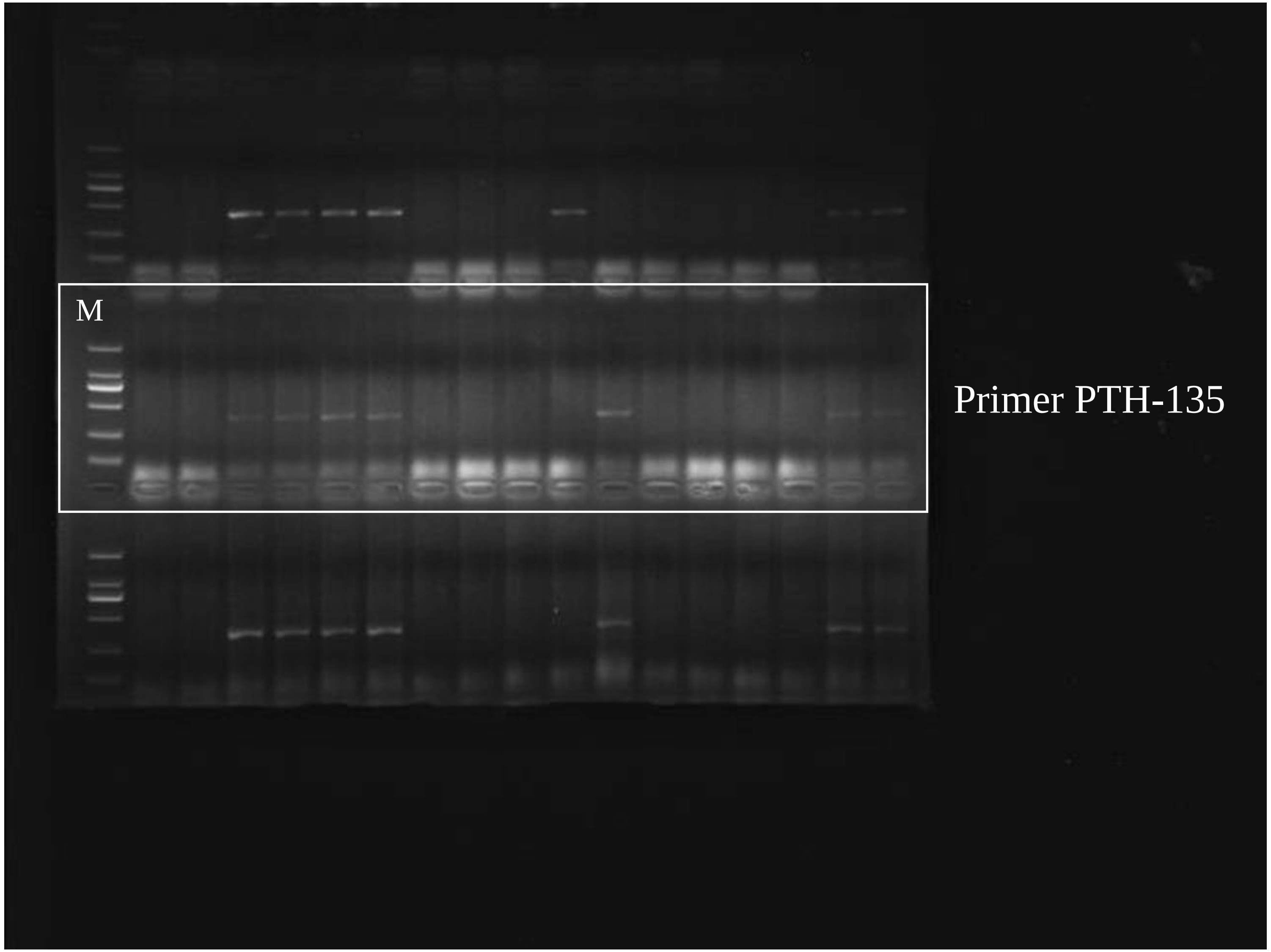

Original gel images of Primer in Fig. 6
