# Supplementary material for: Molecular cytogenetics and development of St-chromosome-specific molecular markers of novel stripe rust resistant wheat–Thinopyrum intermedium and wheat–Thinopyrum ponticum substitution lines
Source: BMC Plant Biol. 2022 Mar 12;22:111. doi: 10.1186/s12870-022-03496-x (PMC8917741; doi:10.1186/s12870-022-03496-x)
Supplement: Supplementary file 9 — Additional file 9: Fig. S6. Uncropped gel images of markers of Fig. 8. [file 12870_2022_3496_MOESM9_ESM.pdf]

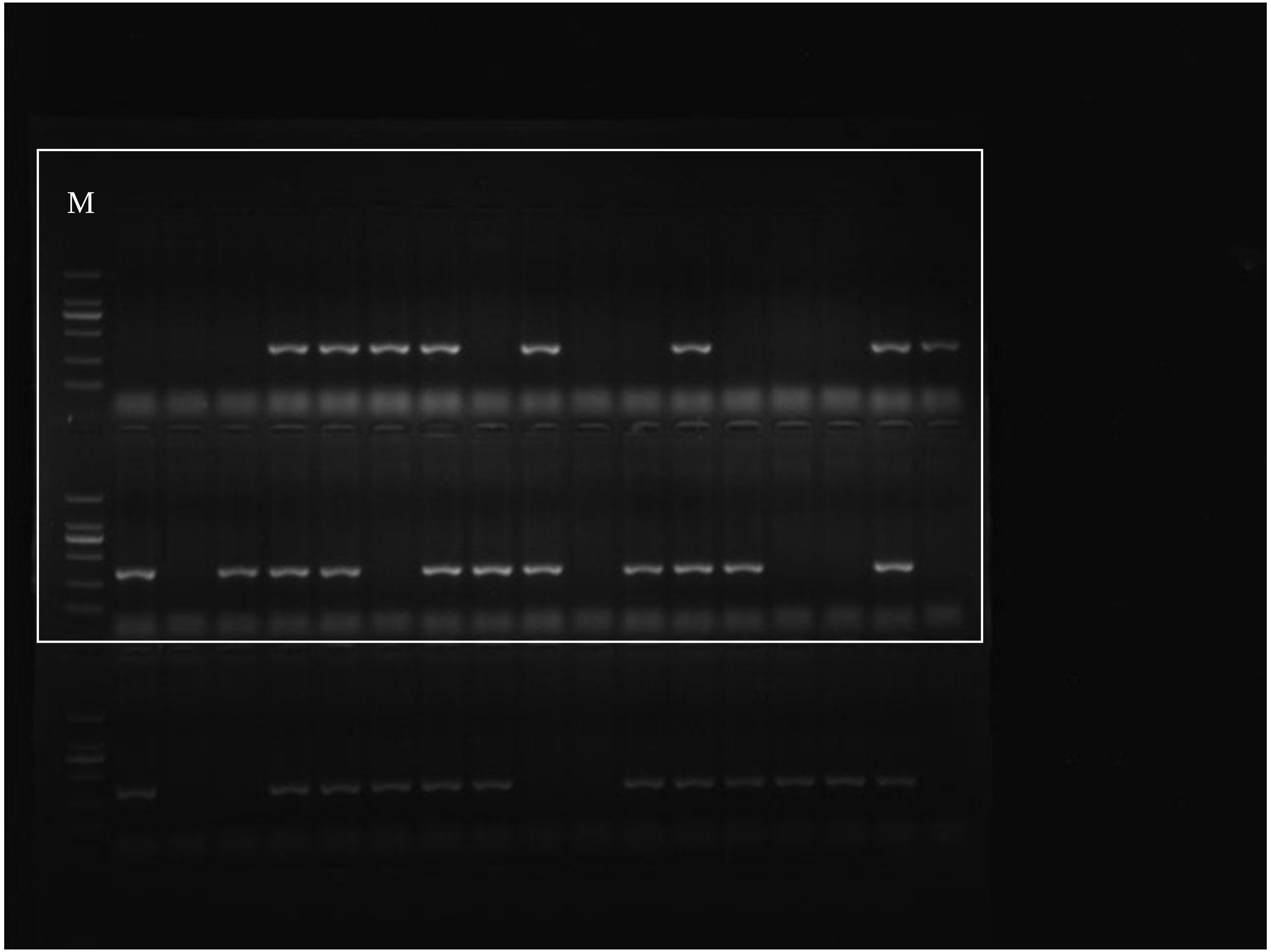


Original gel images of Primer PTH-113 in Fig. 8a (1-34)

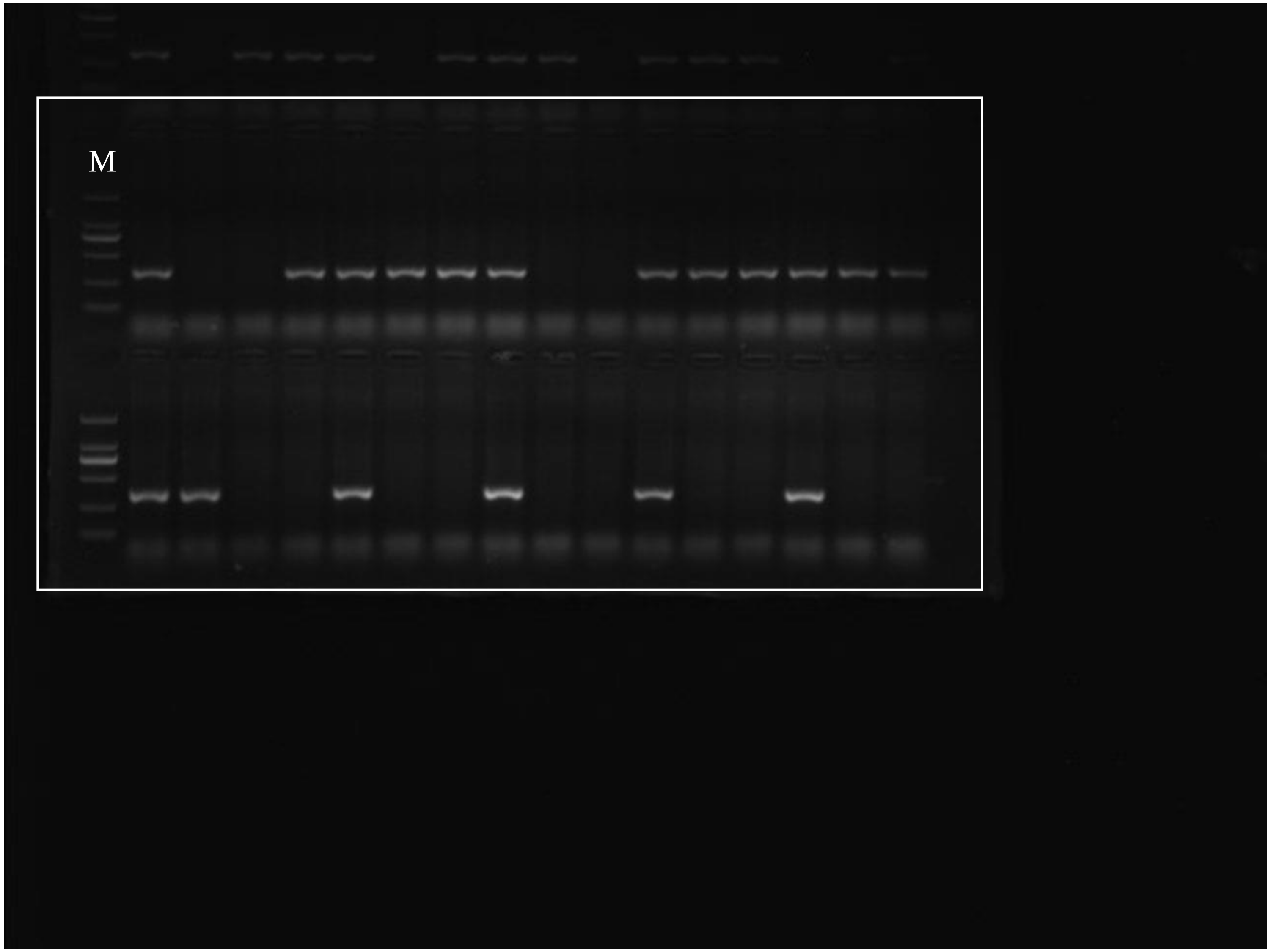


Original gel images of Primer PTH-113 in Fig. 8a (35-67)

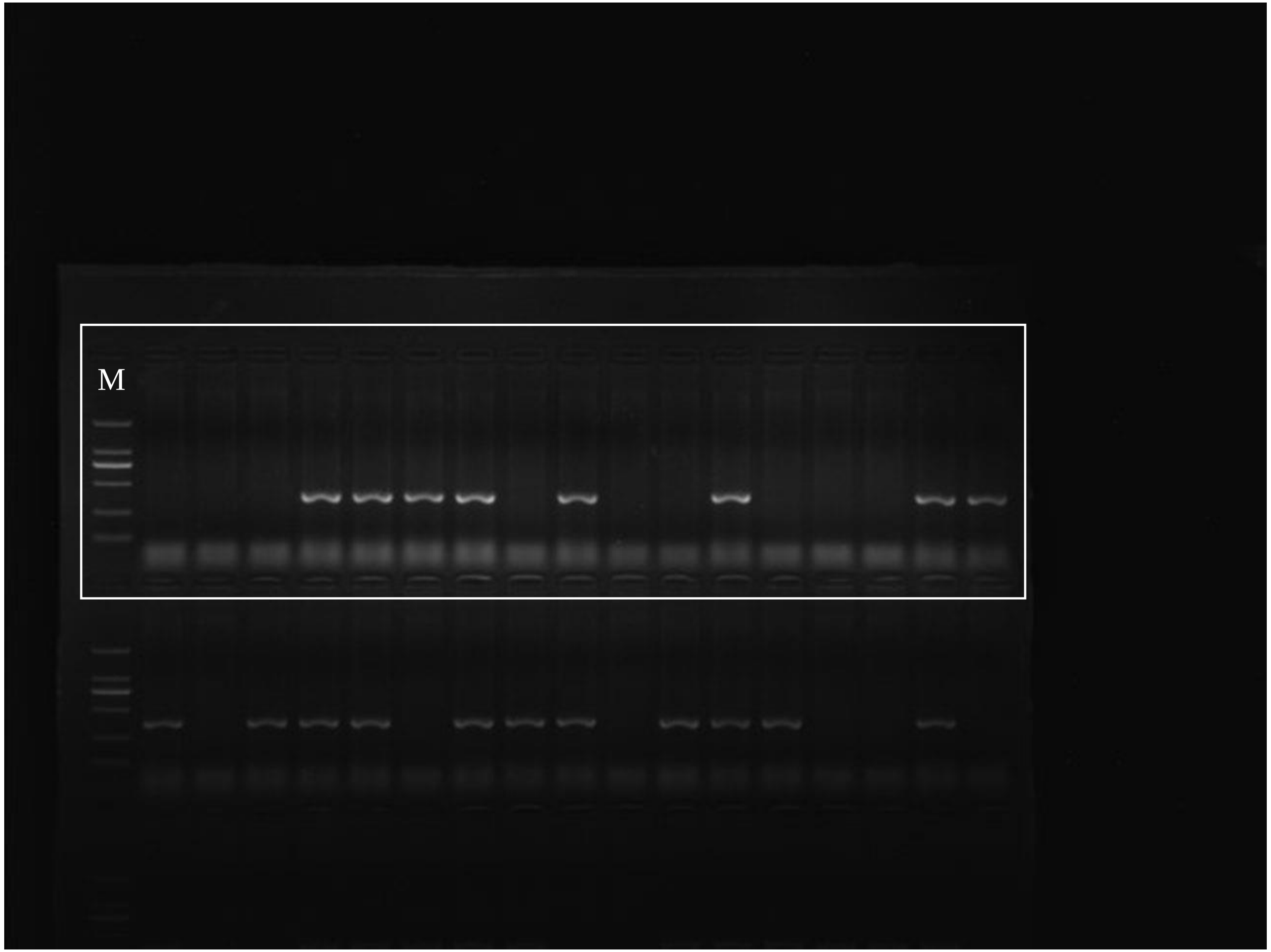


Original gel images of Primer PTH-135 in Fig. 8b (1-17)

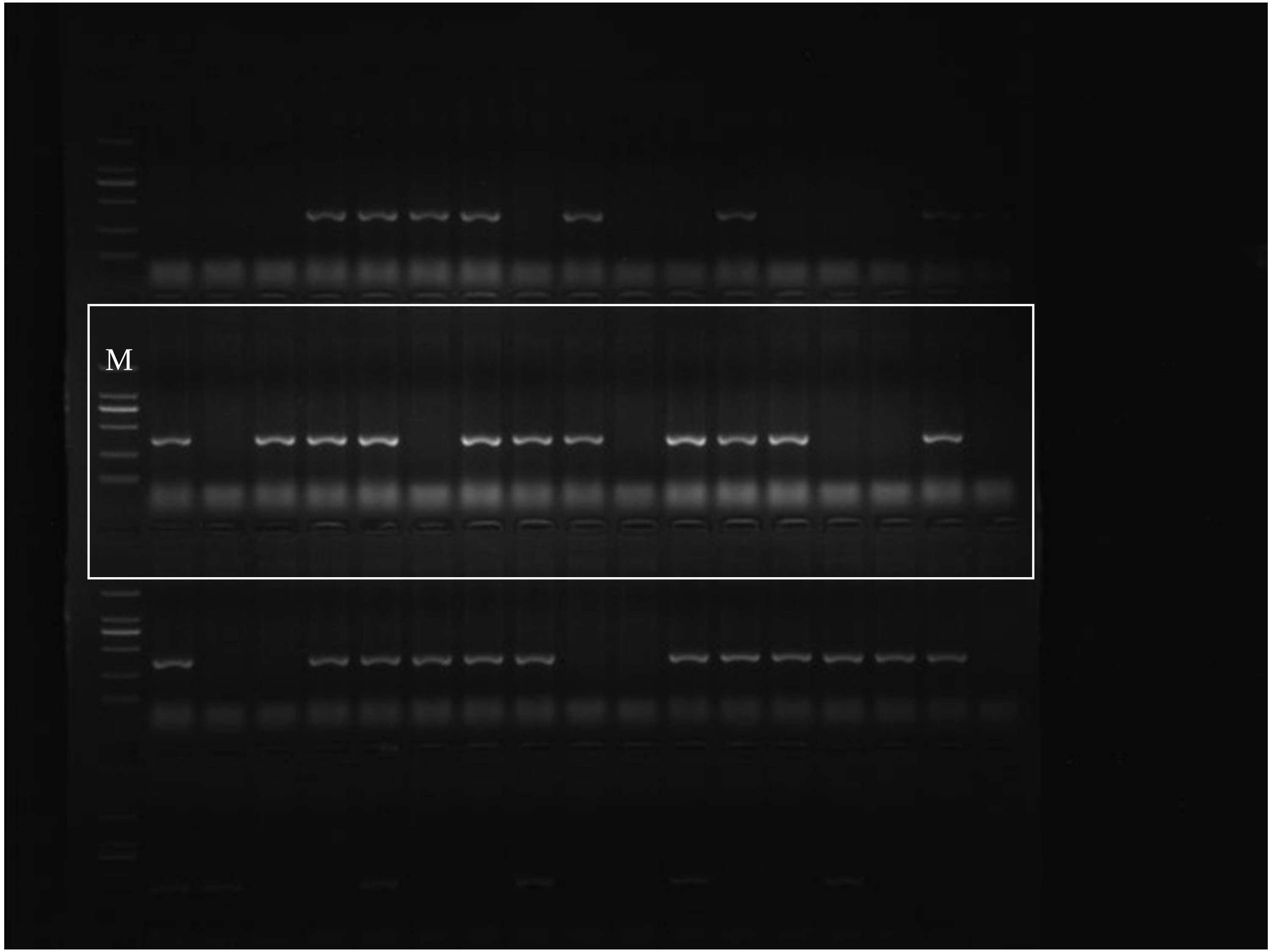


Original gel images of Primer PTH-135 in Fig. 8b (18-34)

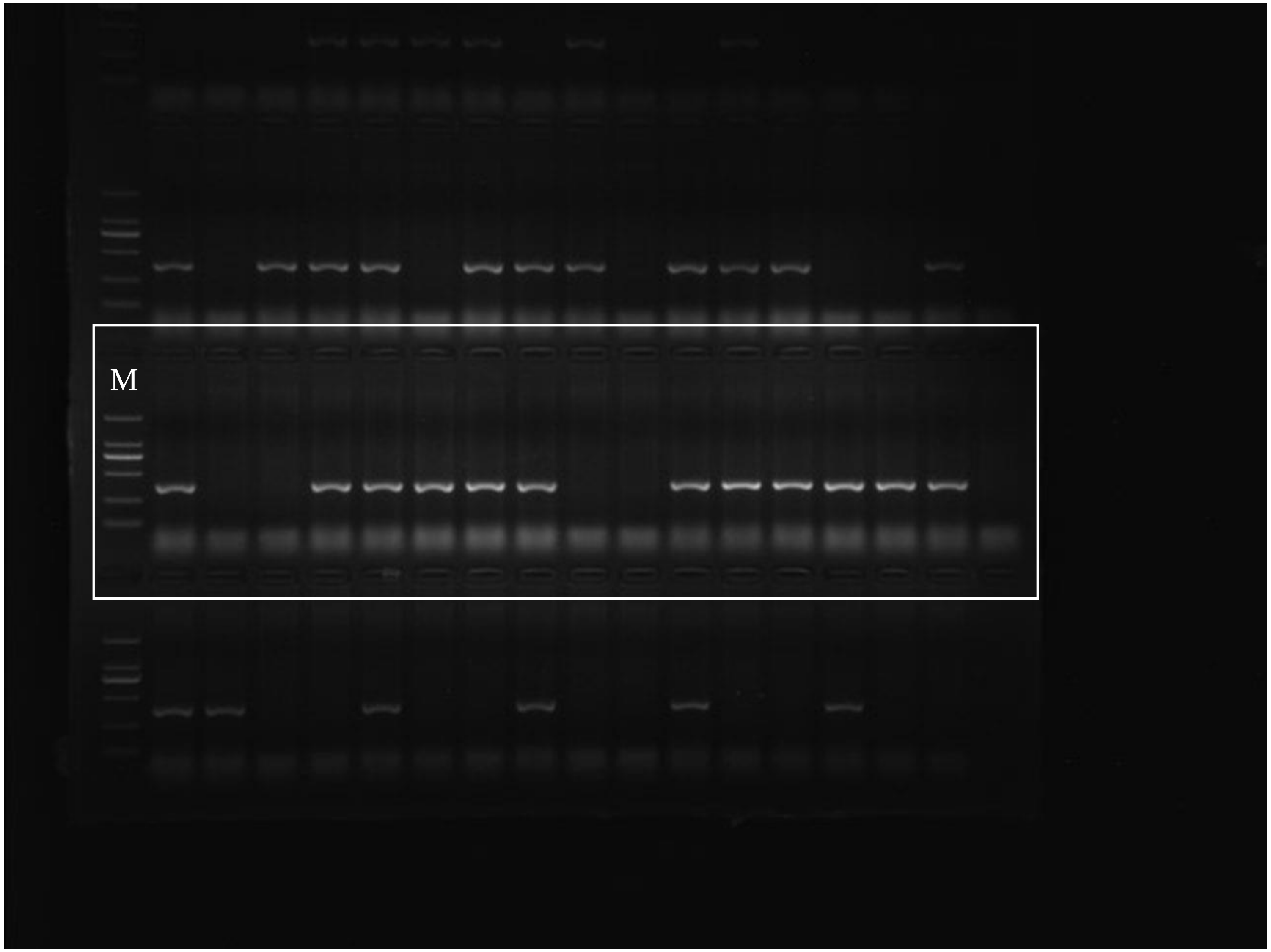


Original gel images of Primer PTH-135 in Fig. 8b (35-51)

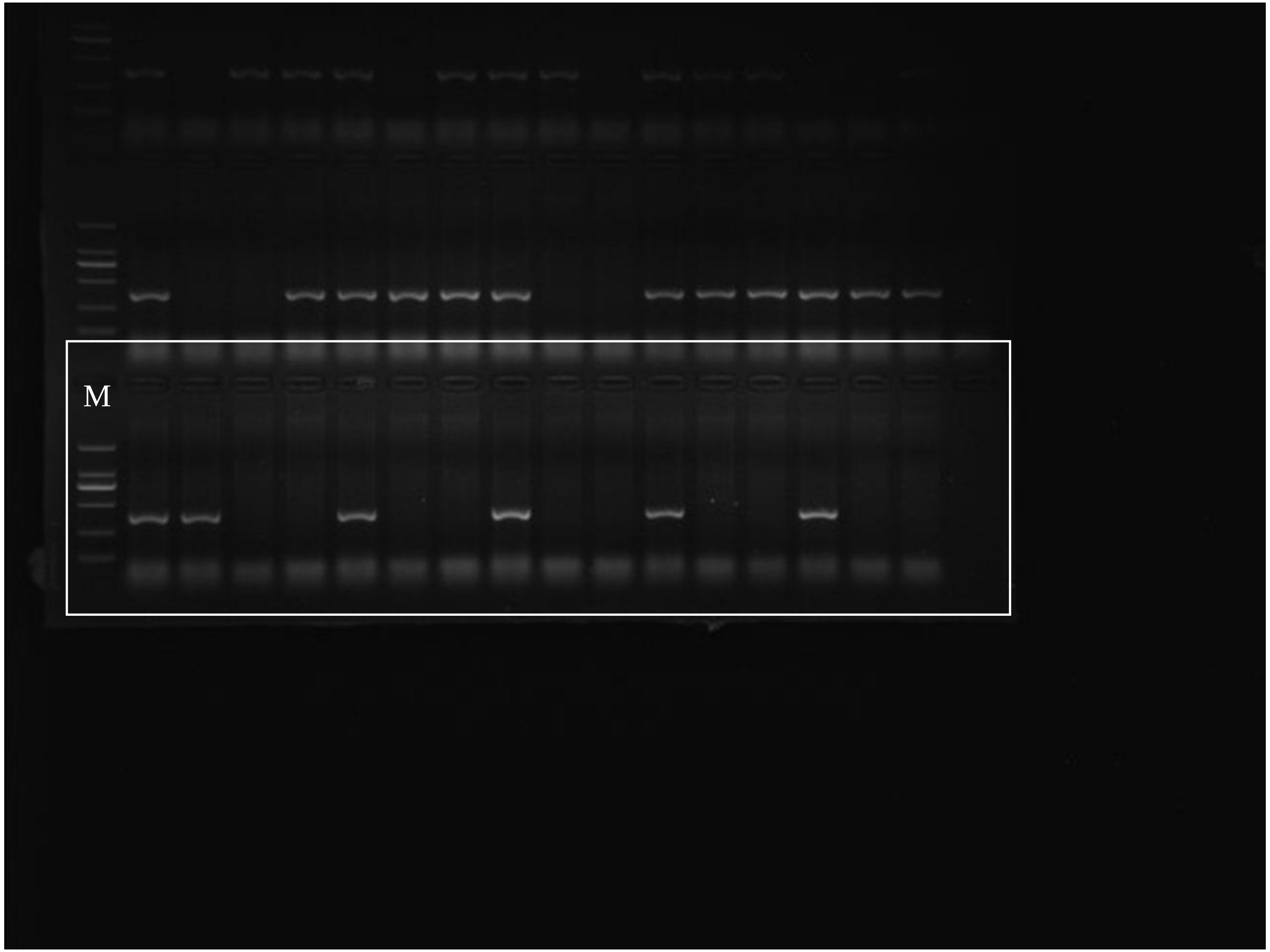


Original gel images of Primer PTH-135 in Fig. 8b (52-67)
